# Transcriptome sequencing of sheep hypothalamic tissue reveals the regulatory role of lncRNA in the mechanism of pubertal estrus initiation

**DOI:** 10.3389/fvets.2025.1594040

**Published:** 2025-09-02

**Authors:** Rong Xuan, Yanan Peng, XinKun Wang, Wei Li, QiaoYan Huang, HuiPing Sun, LeXiao Zhu, RuoHuai Gu, Feng Xing

**Affiliations:** Key Laboratory of Livestock and Forage Resources Utilization around Tarim, Ministry of Agriculture and Rural Affairs, College of Animal Science and Technology, Tarim University, Alar, Xinjiang, China

**Keywords:** lncRNA, estrus initiation, hypothalamic tissue, puberty, Dolang sheep

## Abstract

**Introduction:**

Pubertal estrus in sheep is crucial for reproductive maturation, with the hypothalamus playing a key role in its regulation. While long noncoding RNAs (lncRNAs) have gained attention for their involvement in the nervous system, their specific role in regulating pubertal estrus remains unclear.

**Methods:**

This study performed transcriptome sequencing on hypothalamic tissues from Dolang sheep at pre-pubertal, pubertal, and post-pubertal stages. Identified lncRNAs were characterized based on genomic features, differential expression, potential cis-regulatory targets, and ceRNA relationships.

**Results:**

A total of 1,589 lncRNAs were identified, including 848 known and 741 novel lncRNAs, with intergenic lncRNAs being most abundant. The majority of lncRNAs were 200–2000 nucleotides in length and contained 2–5 exons. A total of 129 differentially expressed lncRNAs were identified, with the highest differential expression between puberty and post-puberty. Cis-regulation analysis suggested that lncRNAs regulate genes involved in estrogen biosynthesis, dopamine transport, glycolipid metabolism, and nervous system development. LncRNAs may also impact reproductive hormone signaling, including the estrogen signaling pathway and gonadotropin-releasing hormone (GnRH) pathways, influencing growth, gonadal development, and sex hormone production. Specifically, ceRNA relationships such as LOC101105119–oar-miR-106b–GNAQ and LOC105607217–oar-miR-370-3p–PRKCA were validated.

**Discussion:**

Overall, this study demonstrates that lncRNAs regulate pubertal estrus initiation through ceRNA networks (e.g., LOC101105119–miR-106b–GNAQ) and hormone signaling, particularly the GnRH pathway, offering potential targets for sheep breeding.

## 1 Introduction

Puberty marks the transition to reproductive maturity, characterized by reproductive system maturation and activation of the hypothalamic–pituitary–gonadal (HPG) axis (1). In females, it is typically indicated by the onset of the estrus cycle, a key marker of reproductive readiness (2). The hypothalamus, as the central regulator of the endocrine system, integrates environmental, metabolic, and hormonal signals to control reproductive hormone secretion via the HPG axis, thereby determining the timing of estrus initiation (3, 4). However, the molecular mechanisms underlying this regulation—particularly the role of long non-coding RNAs (lncRNAs)—remain largely unexplored in sheep.

lncRNAs are RNA molecules longer than 200 nucleotides that do not encode proteins but play important roles in epigenetic, transcriptional, and post-transcriptional regulation (5, 6). Compared with protein-coding mRNAs, lncRNAs exhibit higher spatiotemporal specificity and have been implicated in various reproductive processes, including ovarian development, oocyte maturation, fertilization, and early embryonic development (7–10). Hypothalamus-associated lncRNAs are involved in regulating responses to glucocorticoids, estradiol, and retinoic acid. For example, in Small-tailed Han sheep, the *FecB* gene mutation modulates GnRH secretion and follicle–luteum transition through specific lncRNAs (LINC-219386, IGF2-AS), thereby increasing ovulation rate (11). In goats with divergent fertility, differentially expressed hypothalamic lncRNAs and mRNAs are enriched in *GnRH*, estrogen, and oxytocin signaling pathways, influencing follicular development via key genes such as *NMUR2*, *FEZF1*, and *WT1* (12, 13). Moreover, knockdown of lncRNA Meg3 reduces reproductive hormone levels and delays puberty onset in female rats (14), while high expression of lncRNA AK044061 in arcuate nucleus *AgRP* neurons activates the NF-κB pathway to alter energy metabolism (15), highlighting the critical roles of hypothalamic lncRNAs in both reproductive and metabolic regulation.

Although previous studies have revealed the critical roles of hypothalamic lncRNAs in reproductive regulation in various animal species, systematic investigations during puberty-associated estrus initiation in Xinjiang Dolang sheep are still lacking. The Xinjiang Dolang sheep is a locally adapted breed with strong reproductive performance (16, 17), and recent advances in molecular breeding have markedly improved its reproductive efficiency (17–19). Elucidating the regulatory role of lncRNAs in the hypothalamus during puberty in this breed will provide important insights into the molecular mechanisms of reproductive regulation and identify potential molecular targets for improving fertility. In this study, we performed deep transcriptome sequencing of hypothalamic tissues from pre-pubertal, pubertal, and post-pubertal Dolang sheep to comprehensively identify lncRNAs and their potential target genes involved in estrus initiation. Bioinformatics analyses were then applied to characterize their expression patterns, predict target genes, and explore enriched signaling pathways. These findings will offer new perspectives for understanding the lncRNA-mediated regulatory network underlying estrus initiation in pubertal sheep.

## 2 Materials and methods

### 2.1 Ethics statement

This study was conducted under the supervision and guidance of the Ethics Committee of Tarim University (The ethical approval number is 2024060, Supplementary Figure S1) (16). All experimental participants were required to undergo training and adhere to strict experimental procedures.

### 2.2 Collection of hypothalamic samples, extraction of RNA, and sequencing

This study utilized Dolang sheep from the Experimental Station of Tarim University as a model. The study was conducted from August to September in autumn. Dolang sheep at the prepuberty stage (*n* = 3, 120 days after birth), the puberty stage (*n* = 3, 130 days after birth), and the postpuberty stage (*n* = 3, 150 days after birth) were used in this study. Detailed information on weight, body size, and age can be found in Supplementary Table S1 and Supplementary Figure S2. Pubertal stage classification was performed by observing each ewe at 10:00, 14:00, and 18:00 daily, following criteria described in our previous study (16). Specifically, in the prepubertal stage, ewes remained calm, did not accept mounting by rams, and had dry vulvas; during puberty, ewes displayed restlessness, increased locomotor activity, accepted mounting, and exhibited moist and reddened vulvas with mucus secretion; in the postpubertal stage, ewes again became calm, refused mounting, and the vulvas returned to a dry state. All sheep were in good health and were bred and managed under the same conditions. Samples of hypothalamic tissue were collected from ewes exhibiting signs of puberty for the first time. After intravenous administration of pentobarbital sodium (100 mg/kg) to each sheep, muscle relaxation and cessation of heart and breathing occurred. Subsequently, the sheep were promptly dissected and hypothalamic tissue was harvested. The samples were immediately frozen in liquid nitrogen and stored at −80°C for further analysis. Additionally, hypothalamus samples were collected from pre-pubertal and post-pubertal ewes raised under similar conditions. In total, hypothalamic tissues from nine sheep were collected and analyzed with three biological replicates for each period. The hypothalamus was used to extract total RNA content, using TRIzol reagent (CWBIO, Beijing, China). The purity, concentration, and integrity of the RNA samples were assessed using Nanodrop (Thermo Fisher, USA) and Agilent 2100 (Agilent Technologies, USA). For RNA sample preparation, 1 μg of RNA per sample was utilized as input material. mRNA was isolated from the total RNA using magnetic beads attached with poly-T oligonucleotides. Fragmentation was carried out in NEBNext First Strand Synthesis Reaction Buffer (5X) at high temperature with divalent cations. First-strand cDNA synthesis was performed using random hexamer primers and M-MuLV reverse transcriptase. Subsequently, second-strand cDNA synthesis was conducted using DNA polymerase I and RNase H. The remaining overhangs were converted to blunt ends by exonuclease/polymerase activity. After adenylation of the 3′ end of the DNA fragments, NEBNext adapters with hairpin loop structures were ligated for hybridization preparation. To selectively isolate cDNA fragments of 240 bp in length, library fragments were purified using the AMPure XP system (Beckman Coulter, Beverly, USA). Following this step, 3 μL of USER enzyme (NEB, USA) was used with size-selected adapter-ligated cDNA at 37°C for 15 min followed by incubation at 95°C for 5 min before PCR amplification. PCR amplification involved Phusion High-Fidelity DNA Polymerase along with universal PCR primers and index (X) primers. Finally, PCR products underwent purification through the AMPure XP system and library quality assessment utilizing an Agilent Bioanalyzer 2100 system. Index-encoded samples were clustered on the cBot Cluster Generation System employing TruSeq PE Cluster Kit v4-cBot-HS (Illumina), following manufacturer’s instructions. Upon cluster generation completion; library preparations were sequenced on the Illumina platform provided by Biomarker Technologies resulting in paired-end reads being generated.

### 2.3 Alignment of sequencing data to the sheep reference genome and the identification and classification of lncRNAs

The quality of the raw sequencing data was assessed using FastQC software (version 0.11.9) (20). Sequencing adapters and low-quality sequences were removed using Trimmomatic software (version 0.39) (21). The sheep reference genome index was generated with HISAT2 software (version 2.2.1) (22), and the sequencing reads were aligned to the sheep reference genome to calculate the read alignment rate. The NCBI RefSeq assembly number for the sheep reference genome is GCF_016772045.2. Transcripts were assembled using StringTie software (version 2.2.0) (23), and transcripts from all samples were obtained using the StringTie merge function, which resulted in new annotation files for quantitative transcript analysis. Spliced transcripts were compared with the sheep genome annotation file using GffCompare software (version 0.12.6) (24). Transcripts categorized as “u,” “x,” “i,” “j,” or “o” were selected for downstream analysis screening (25). The screening criteria for novel lncRNAs were set as length > 200 and coverage > 1. Subsequently, the newly assembled transcripts were utilized to predict their protein coding ability using four software tools: CPC2 (version 2) (26), CNCI (version 2) (27), PLEK (version 1.2) (28), and CPAT (version 1.2) (29). The final novel lncRNA was determined by taking the intersection of the prediction results from all four software tools. Furthermore, the FEELnc software (version 0.2.1) (30) was employed to classify the identified lncRNAs into four types based on their positional relationship with adjacent protein-coding genes: intergenic, intronic, sense, and antisense lncRNAs.

### 2.4 Differential expression analysis

First, the data was filtered using RUVseq software (version 1.30.0) (31). lncRNAs with FPKM ≥ 0.5 were retained for principal component analysis (PCA) of all samples. Differential expression analysis was performed using edgeR software (version 3.38.4) (32). lncRNAs were identified as differentially expressed when the conditions log2Foldchange ≥ 1 and false discovery rate (FDR) < 0.05 were met. The expression pattern changes of all differentially expressed lncRNAs were displayed by heat maps. Differentially expressed lncRNAs were clustered according to their expression levels using the R package TCseq software (version 1.20.0) (33). The corresponding miRNAs and their target genes were screened. Gene Ontology (GO) functional annotation and Kyoto Encyclopedia of Genes and Genomes (KEGG) pathway enrichment analysis were performed on the target genes of each cluster.

### 2.5 Analysis of the cis-regulatory functions of differentially expressed lncRNAs

Cis-regulatory functions pertain to the activation of transcription and regulation of expression in neighboring mRNAs by noncoding RNAs. In this study, BEDTools software (version 2.30.0) (34) was utilized to identify protein-coding genes located within 100 kb upstream and downstream of lncRNAs. Furthermore, GO functional annotation and KEGG pathway enrichment analysis were conducted based on the genomic location annotation of lncRNAs.

### 2.6 Analysis of competing endogenous RNA (ceRNA) relationships

Mature sheep miRNA sequence files were obtained from the miRBase website (35). The miRanda software (version 3.3a) (36) was utilized to predict the target relationships between miRNA and lncRNA, as well as miRNA and mRNA. Target relationships with scores ≥ 150 and energy < −10 were selected for further analysis. Subsequently, a miRNA-lncRNA-mRNA (protein coding) ceRNA relationship network was constructed using Cytoscape software (version 3.8.0) (37) in combination with the previously calculated target relationships.

### 2.7 Co-expression analysis of differentially expressed lncRNAs and protein-coding genes

lncRNAs and protein-coding transcripts with FPKM ≥ 0.5 were screened separately. The R package psych (version 2.2.3) was utilized to compute the Pearson correlation between differentially expressed lncRNAs and protein-coding genes, as well as to conduct significance tests. Protein-coding genes exhibiting absolute correlation values ≥0.7 and a *p* value < 0.05 with lncRNA were selected for GO functional annotation and KEGG pathway enrichment analysis.

### 2.8 Performing GO function annotation and KEGG pathway enrichment analysis

The R package clusterProfiler (version 4.4.4) (38) was utilized for conducting GO annotation and KEGG pathway enrichment analysis on the selected target genes. Sheep genes annotated with the GO and KEGG databases were employed as background genes for enrichment analysis, and only GO terms or KEGG pathways with adjust *p*-values (*p*.adj) < 0.05 were considered. Based on the *p*.adj value, the top 15 GO terms or KEGG pathways were chosen for presentation.

### 2.9 Protein–protein interaction (PPI) network analysis

The gene names selected were submitted to the STRING website^1^ (39), and the relationships with a combined score ≥ 0.4 were filtered to obtain the PPI network. The node degree was calculated using the Cytoscape built-in software NetworkAnalyzer, and the network hub genes were selected based on the node degree.

### 2.10 Dual-luciferase reporter assay

miRanda software (version 3.3a) was used to predict the miRNA binding site information of target genes. Wild-type plasmids, mutant plasmids, and positive plasmids of the six transcripts were constructed using the pmirGLO vector. HEK-293 T cells were used for the dual-luciferase reporter assay because primary hypothalamic cells from sheep were not available at the time of the experiment. HEK-293 T cells exhibit high transfection efficiency, stable growth characteristics, and low endogenous expression of the tested miRNAs, minimizing background noise and ensuring reliable detection of reporter activity (40, 41). Empty vector, wild-type, mutant plasmids, and positive plasmids were co-transfected with oar-miR-106b mimics or oar-miR-370-3p mimics into HEK-293 T cells. After 48 h, the firefly and Renilla luciferase activities were measured using a dual-luciferase reporter gene kit (Promega) on a fluorescence luminescence detection instrument (GloMax® 20/20 luminometer (Promega)). The sequences of miRNA mimics and eukaryotic expression vectors are shown in Supplementary Figure S3. Three biological replicates were established for each group.

### 2.11 CeRNA relationship verification

The pcDNA3.1 vector (Sigma-Aldrich) was used to construct the overexpression vectors of *GNAQ* (XM_015093177.3), LOC101105119 (XR_009597486.1), *PRKCA* (XM_060395902.1), and LOC105607217 (XR_006059048.1), oar-miR-106b mimics and oar-miR-370-3p mimics were synthesized by Ribobio Biotech. Cell transfection experiments were divided into the following groups: empty pcDNA3.1 vector without sequence insertion (NC(pcDNA3.1)); gene overexpression vector (gene); lncRNA overexpression vector (lncRNA); miRNA control group (NC (MIMICS)); miRNA overexpression group (miRNA); NC (pcDNA3.1 + MIMICS); miRNA and lncRNA co-overexpression group (miRNA + lncRNA); miRNA, lncRNA, and gene co-overexpression group (miRNA + lncRNA + gene). The above vectors were transfected into HEK-293 T cells. The green fluorescent protein (GFP) fragment was inserted into the pcDNA3.1 overexpression vector (*GNAQ*, LOC101105119, *PRKCA*, and LOC105607217), and the number of cells expressing GFP was observed by fluorescence microscope (Keyence Biorevo BZ-9000) to evaluate the transfection efficiency of the overexpression vector in HEK-293 T cells after 48 h cell transfection. Cell count analysis was performed using ImageJ 1.48 software. Transfection efficiency was determined by quantifying the percentage of GFP+ cells within the total (DAPI+) population. The expression levels of *GNAQ* and *PRKCA* in the above different groups were detected by real-time quantitative reverse-transcription PCR (RTqPCR) 48 h after cell transfection. Genes and lncRNA overexpression vectors were transfected using Lipofectamine 3000 kit (Thermo Fisher Scientific). Mimic of miRNA and its control group were transfected using Lipofectamine™ RNAiMAX (Thermo Fisher Scientific).

### 2.12 Real-time quantitative reverse-transcription PCR (RTqPCR)

To assess the accuracy of RNA sequencing, 10 differentially expressed lncRNAs were randomly chosen for RT-qPCR analysis. The primers were designed using NCBI Primer-BLAST (42). Total RNA was extracted from hypothalamic tissue using the Trizol method. Reverse transcription and PCR amplification were carried out using the One-Step TB Green PrimeScript RT-PCR Kit (Takara, Beijing, China). Quantification was performed with a LightCycler® 96 instrument (Roche, Switzerland). The primer amplification efficiency was determined by establishing a standard curve. After assessing stability with NormFinder (version 5.0) (43), the geometric mean of two reference transcripts (XM_015102346.4 and XM_015094093.4) was selected as the normalization factor. Relative gene expression was calculated using the modified Pfaffl method (44) (for detailed calculation steps, see: https://toptipbio.com/qpcr-multiple-reference-genes/). The primer sequences and efficiencies are provided in Supplementary Table S2.

### 2.13 Statistical analysis

The significance of differences between the experimental group and the control group was tested using the Student’s t-test, with a threshold of *p* < 0.05 indicating a significant difference. Gene expression levels at different developmental stages were analyzed using Tukey’s honestly significant difference test. Unless otherwise specified, the plots presented in this work were generated using R software (Version 4.4.1).^2^

## 3 Results

### 3.1 Results of basic statistical analysis of sequencing data

**Figure 1:**
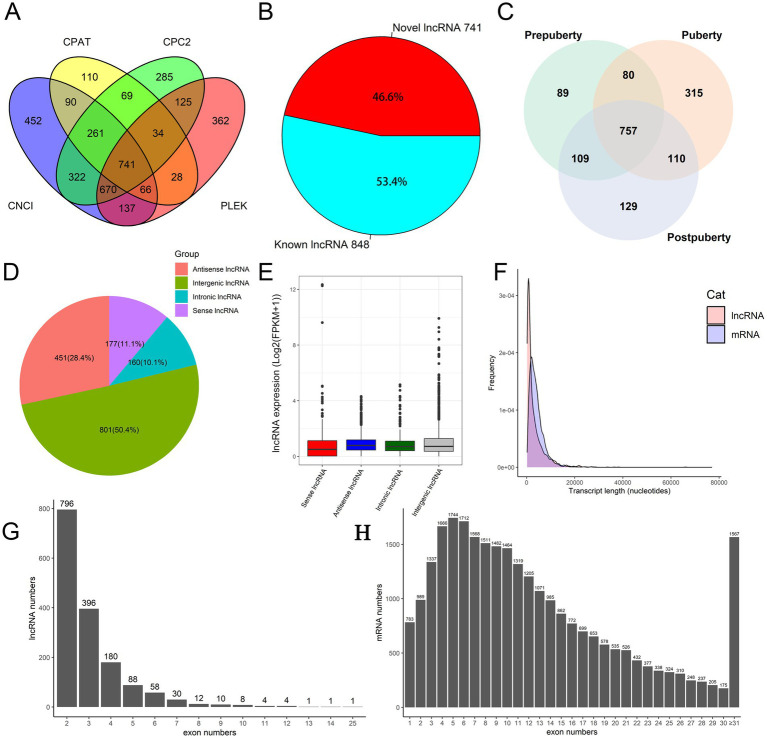
Identification and characterization of lncRNAs in hypothalamic tissue. **(A)** Coding ability of novel lncRNAs. **(B)** The number of known and novel lncRNAs. **(C)** Venn plot of lncRNA expression in the prepuberty, puberty, and postpuberty. **(D)** Classification and statistical analysis of lncRNAs based on genome location information. **(E)** Logarithmic expression levels (log(FPKM+1)) of four types of lncRNA. **(F)** The distribution of length in lncRNA and mRNA. **(G)** Statistics of lncRNA exon numbers. **(H)** Statistics of mRNA exon numbers.

Statistical analysis of the sequencing data revealed that 24,405,562 ± 628,416, 23,243,346 ± 1,957,677, and 23,679,907 ± 1,788,387 clean reads were obtained from the sequencing libraries of prepubertal, pubertal, and postpubertal stages, respectively. The alignment results showed that the alignment rate of each sequencing library was ≥91% (Supplementary Table S3). The original sequencing data has been submitted to the NCBI GEO database (GEO accession number: GSE273981). Combined with the annotation of the sheep reference genome and the structural and functional characteristics of lncRNAs, this study identified 848 known lncRNAs and 741 novel lncRNAs (Figures 1A,B and Supplementary Table S4). The expression of lncRNA in each period was counted (FPKM ≥ 0.5): the number of lncRNAs expressed in puberty was highest (1,262), the number expressed in pre-puberty was lowest (1,035), and 757 lncRNA species were expressed in all three periods (Figure 1C). This indicates that lncRNA expression is more active during puberty. Furthermore, certain lncRNAs are expressed uniquely in each stage, indicating the stage-specific expression of lncRNAs during different puberty stages of the hypothalamus.

### 3.2 Analysis of the exon number and length characteristics of lncRNA

The classification of lncRNAs based on their genomic location revealed that the number of intergenic lncRNAs exceeded that of intronic lncRNAs (Figure 1D and Supplementary Table S5). This suggests that intergenic lncRNAs may exhibit higher transcription efficiency and stability, leading to their accumulation at higher levels in cells. In contrast, intronic lncRNAs may be less abundant due to degradation during post-transcriptional processing. The Figure 1E shows the relative logarithmic expression of four different types of lncRNAs. Length distribution statistics (Figure 1F) indicated that the average length of mRNA was approximately 2000 nucleotides, while the length of lncRNAs ranged from hundreds to tens of thousands of nucleotides, with most falling within the range of 200–2,000 nucleotides. Analysis of transcript exon numbers showed that a majority of lncRNAs had 2–5 exons (Figure 1G), whereas mRNAs typically had between 1 and 22 exons (Figure 1H). Furthermore, the overall expression level of mRNA was found to be higher than that of lncRNA (Supplementary Figure S4). In summary, this study observed greater overall expression levels, lengths, and numbers of exons for mRNA in sheep hypothalamic tissue compared to those for lncRNA.

### 3.3 Results of differential expression analysis

**Figure 2:**
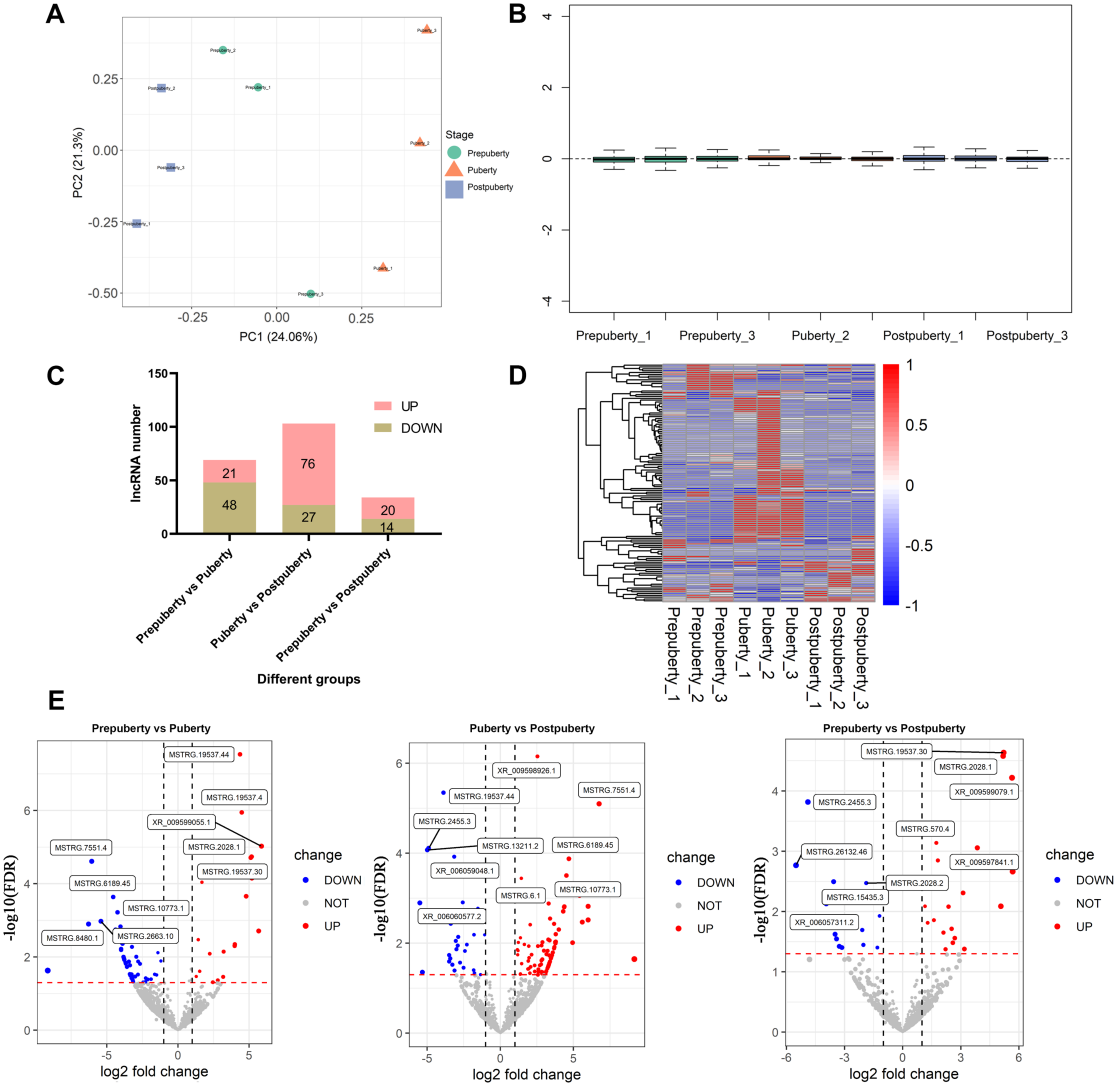
Analysis of differentially expressed lncRNAs at various stages of puberty. **(A)** Principal component analysis of different samples. **(B)** Relative log expression (log FPKM) of different samples. **(C)** Statistical results of differentially expressed lncRNAs between different comparison groups. **(D)** Heat map of differentially expressed lncRNAs. **(E)** Volcano plot depicting the differentially expressed lncRNAs across various comparison groups.

The principal component analysis (PCA) revealed (Figure 2A) that, following sample correction, the hypothalamic samples from the same period clustered together, while samples from different periods were scattered. The sample expression box plot provided an intuitive display of expression distribution among hypothalamic samples, indicating proper normalization and removal of expression outliers (Figure 2B). Differential expression analysis results identified a total of 129 differentially expressed lncRNAs in the three comparison groups (Supplementary Table S6). Notably, the comparison between puberty and postpuberty exhibited the largest number of differentially expressed lncRNAs (Figure 2C). The heat map illustrated the expression changes of these 129 lncRNAs across the three developmental stages (Figure 2D), while the volcano map depicted the top five up-regulated and down-regulated lncRNAs in the three-group comparison (Figure 2E). In summary, these findings demonstrate that transcriptome levels of lncRNAs in the hypothalamus undergo changes at different stages of puberty, reflecting stage-specificity in lncRNA expression within hypothalamic tissue. Additionally, RTqPCR results for 10 randomly selected lncRNAs are presented in Supplementary Figure S5. Importantly, there was a significant positive correlation between RNA-Seq results (log2 fold change) and RTqPCR results (log2 fold change), with a correlation value of 0.89 and *p* value < 0.01. This indicates high accuracy in both RNA sequencing and differential expression analysis results.

### 3.4 Results of lncRNA cis-regulatory function analysis

**Figure 3:**
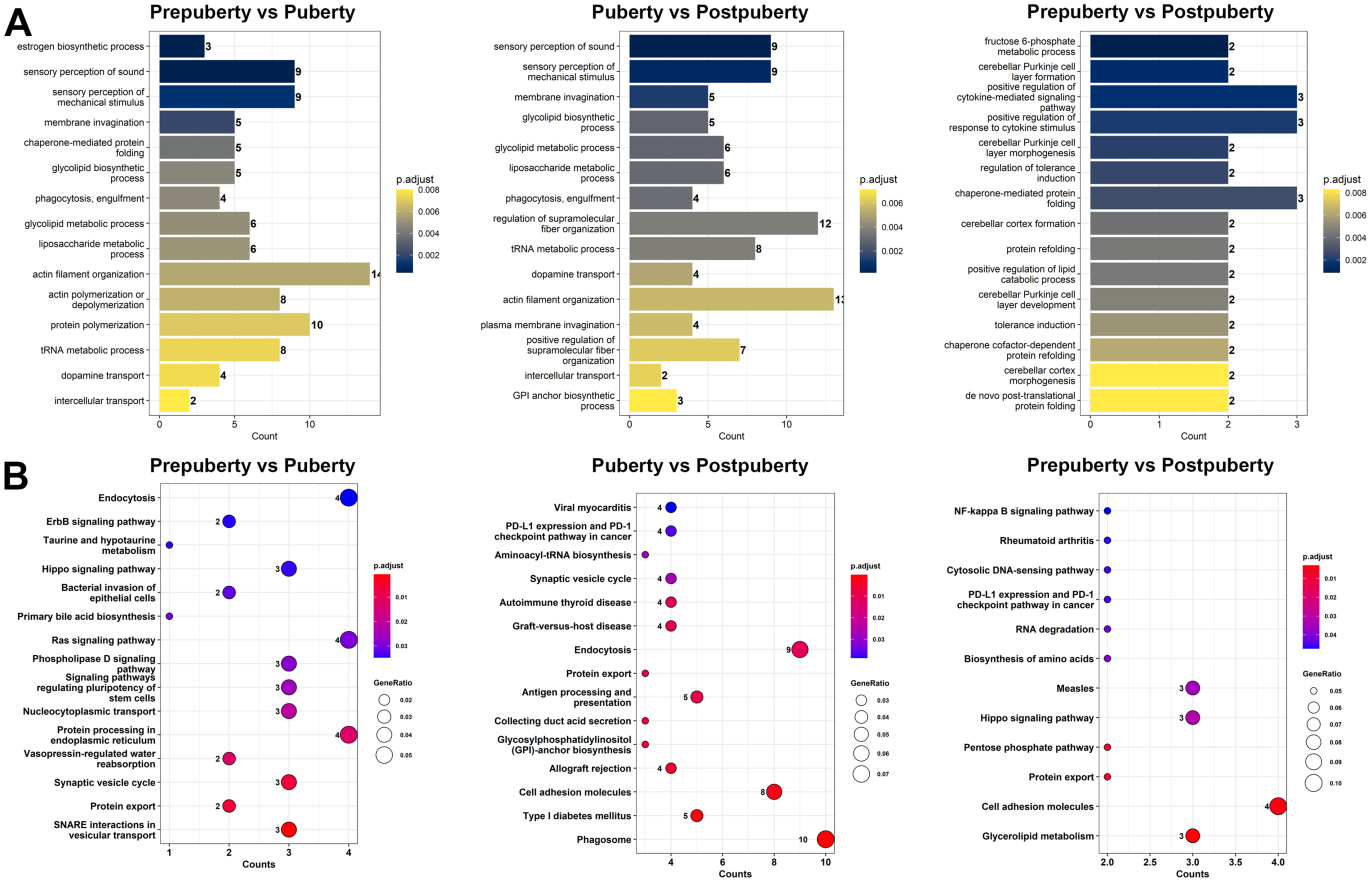
GO functional annotation and KEGG pathway enrichment analysis of lncRNA target genes for cis-regulatory function. **(A)** GO functional annotation of cis-acting target genes of differentially expressed lncRNAs in the three comparison groups. **(B)** KEGG pathway enrichment analysis of cis-acting target genes of differentially expressed lncRNAs in the three comparison groups.

In this study, a total of 733 cis-regulatory relationships were identified (Supplementary Table S7), comprising 88 lncRNA genes (101 variant lncRNA transcripts) and 313 protein-coding genes (630 variant mRNA transcripts). The GO analysis results revealed the significant enrichment of GO terms in different comparisons. In the Prepuberty vs. Puberty group, enriched GO terms included estrogen biosynthetic process, dopamine transport, glycolipid metabolic process, and liposaccharide metabolic process (Figure 3A). In the Puberty vs. Postpuberty group, significantly enriched GO terms comprised sensory perception of sound, sensory perception of mechanical stimulus, and membrane invagination. Lastly, in the Prepuberty vs. Postpuberty group, enriched GO terms related to brain development such as cerebellar Purkinje cell layer formation, cerebellar Purkinje cell layer morphogenesis, and cerebellar cortex formation were observed. Additionally, KEGG signaling pathway analysis showed significant enrichment of pathways related to cell growth and differentiation including ErbB signaling pathway, Hippo signaling pathway, and Synaptic vesicle cycle pathways (Figure 3B).

**Figure 4:**
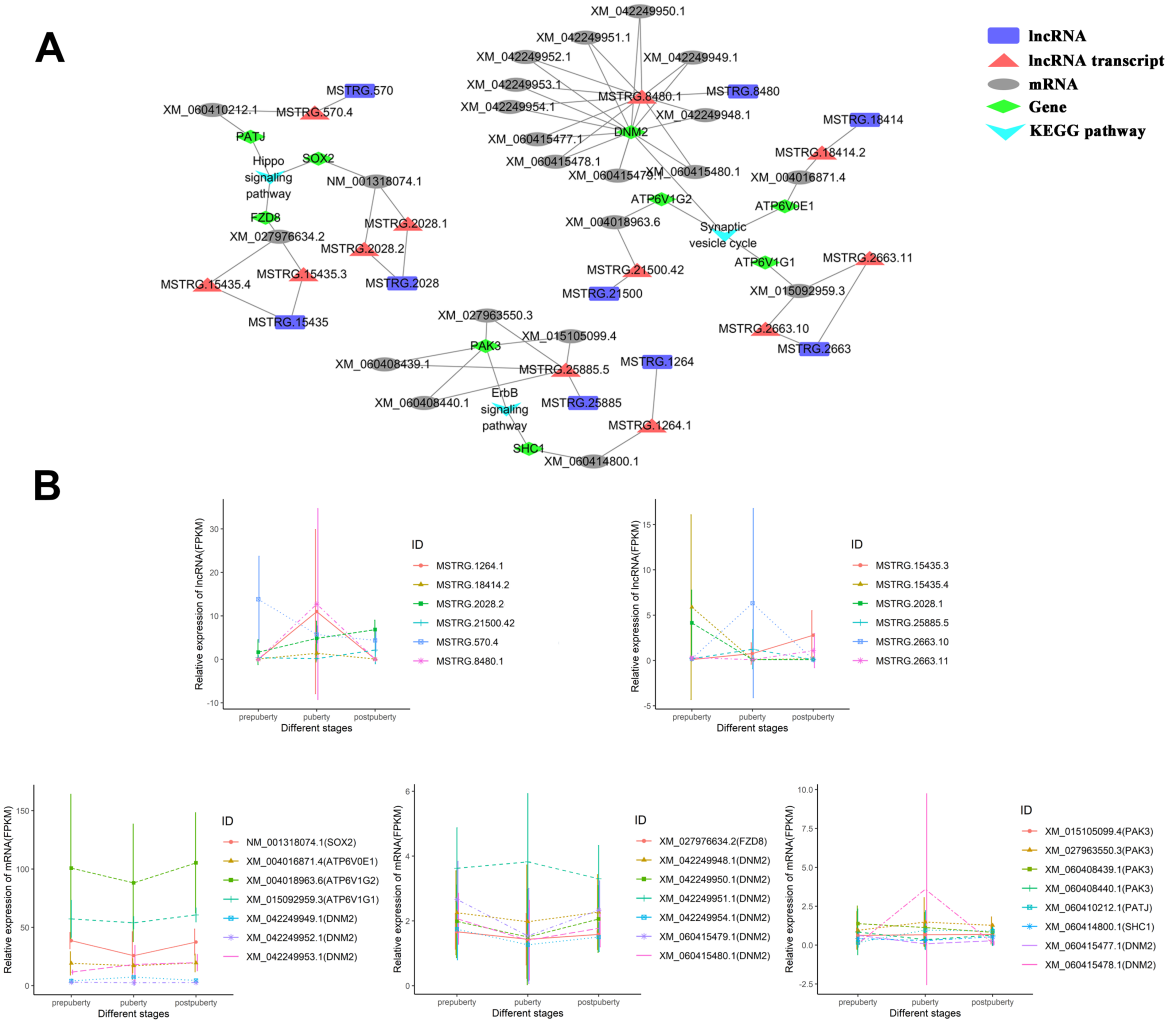
lncRNA-mRNA cis-regulatory network. **(A)** The lncRNA-mRNA cis-regulatory network associated with the ErbB signaling pathway, hippo signaling pathway, and synaptic vesicle cycle pathways. **(B)** Line graph depicting the expression levels of mRNA and lncRNA in the cis-regulatory network.

This study has established the lncRNA-mRNA cis-regulatory network associated with the aforementioned three signaling pathways (Figure 4A). Specifically, there are five lncRNA transcripts (derived from MSTRG.15435, MSTRG.2028, and MSTRG.570 lncRNA genes) that exert cis-regulatory effects on the Hippo signaling pathway by influencing *PATJ*, *SOX2*, and *FZD8*. Among them, lncRNA transcripts MSTRG.15435.4, MSTRG.570.4, and MSTRG.2028.1 exhibit high expression levels during prepuberty, while MSTRG.2028.2 and MSTRG 15435.3 are highly expressed at postpuberty (Figure 4B). There are five lncRNA transcripts (derived from MSTRG.18414, MSTRG.21500, MSTRG.2663, and MSTRG.8480 lncRNA genes) that regulate the synaptic vesicle cycle by affecting *ATP6V0E1*, *ATP6V1G1*, and *DNM2*. Additionally, there are two lncRNA transcripts (derived from MSTRG.1264 and MSTRG.25885 lncRNA genes) that regulate the ErbB signaling pathway by affecting *SHC1* and *PAK3*. In summary, the aforementioned lncRNAs may play a role in regulating neural cell development, signal transduction, and hormone secretion through cis action.

### 3.5 Analysis of lncRNA-miRNA-mRNA ceRNA relationship in hypothalamic tissue

Using miRanda software, we obtained 1,531 miRNA-lncRNA and 78,542 miRNA-mRNA targeting relationships (Supplementary Table S8). The GO analysis results (Supplementary Figure S6A and Supplementary Table S8) indicated a significant enrichment of GO terms related to the development of the nervous system, such as regulation of neuron projection development, dendrite development, and regulation of neurogenesis. Additionally, there was a significant enrichment of GO terms related to neurotransmitters or neural signaling, including cytosolic transport, vesicle transport along the microtubule, anterograde axonal transport, axo-dendritic transport, and vesicle-mediated transport in the synapse. This suggests that lncRNA may play a role in promoting neuronal interaction by regulating gene expression, participating in cell signaling pathways, and influencing cell morphology. The KEGG results revealed (Supplementary Figure S6B and Supplementary Table S8) that signaling pathways associated with the endocrine system were significantly enriched. These included pathways related to parathyroid hormone synthesis, secretion and action, GnRH secretion, oxytocin signaling pathway, cortisol synthesis and secretion, renin secretion, estrogen signaling pathway, and insulin secretion. Additionally, signaling pathways related to signal transduction such as Rap1 signaling pathway, calcium signaling pathway, MAPK signaling pathway, PI3K-Akt signaling pathway, and cAMP signaling pathway were also found to be significantly enriched.

### 3.6 The regulatory role of hormone-related lncRNA in the hypothalamus

**Figure 5:**
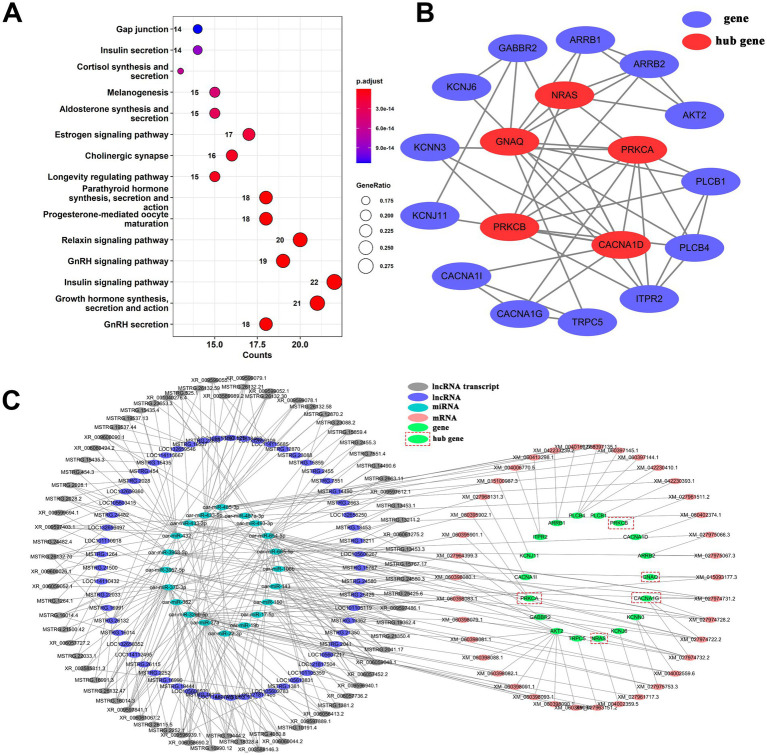
The ceRNA network of lncRNA-miRNA-mRNA in the signaling pathway of GnRH secretion. **(A)** A dotplot analysis of KEGG signaling pathways revealed significant enrichment in the regulation of estrus and reproductive function. **(B)** Protein and protein interaction network diagram of genes enriched in the signaling pathway for GnRH secretion. **(C)** The GnRH secretion ceRNA network.

Further analysis of the ceRNA relationship of lncRNA-miRNA-mRNA revealed that a total of 78 genes were associated with hormones. Pathway enrichment analysis of these genes indicated (Figure 5A) significantly enriched signaling pathways related to the regulation of estrus and reproductive function, such as GnRH secretion, growth hormone synthesis, secretion and action, insulin signaling pathway, GnRH signaling pathway, and estrogen signaling pathway. Among them, 18 genes were enriched in GnRH secretion. PPI results (Figure 5B) identified *NRAS*, *GNAQ*, *PRKCA*, *PRKCB*, and *CACNA1D* as hub genes in the PPI network. In the GnRH secretion ceRNA network (Figure 5C), there are 75 lncRNA transcripts, 60 lncRNA genes, 38 mRNA transcripts, 18 genes, and 20 miRNAs. The diagram of the GnRH secretion pathway illustrates the expression of enriched genes (Supplementary Figure S7A). Among the identified genes, *GNAQ* and *ARRB1* exhibit upregulation in prepuberty but downregulation during puberty and postpuberty. The targeted relationship prediction suggests that lncRNA transcripts MSTRG.15767.17, MSTRG.24580.3, MSTRG.25425.6, and XR_009597486.1 may modulate *GNAQ* expression through oar-miR-106b. Specifically, XR_009597486.1 shows high expression in prepuberty, while MSTRG.25425.6 and MSTRG.15767.17 are highly expressed during puberty, and MSTRG.24580.3 is highly expressed during both puberty and postpuberty (Supplementary Figure S7B). In addition, this study also constructed a ceRNA network for growth hormone synthesis, secretion, and action (Supplementary Figure S8). The network consisted of 57 lncRNA transcripts, 47 lncRNA genes, 47 mRNA transcripts, 21 genes, and 22 miRNAs. A total of 10 genes (*ADCY1*, *ADCY6*, *ADCY7*, *ADCY9*, *PLCB1*, *AKT2*, *GSK3B*, *PRKCB*, *PRKACB*, and *PRKCA*) were identified as hub genes of the PPI network (Supplementary Figure S8B). In summary, the above analysis shows that during the reproductive process of puberty, lncRNAs can regulate the expression of reproductive-related hormone signaling pathways such as GH and GnRH through miRNAs. This regulation in turn affects growth, gondal development, and sex hormone production to ensure normal reproductive maturity and physical development.

### 3.7 GnRH secretion network ceRNA relationship verification results

**Figure 6:**
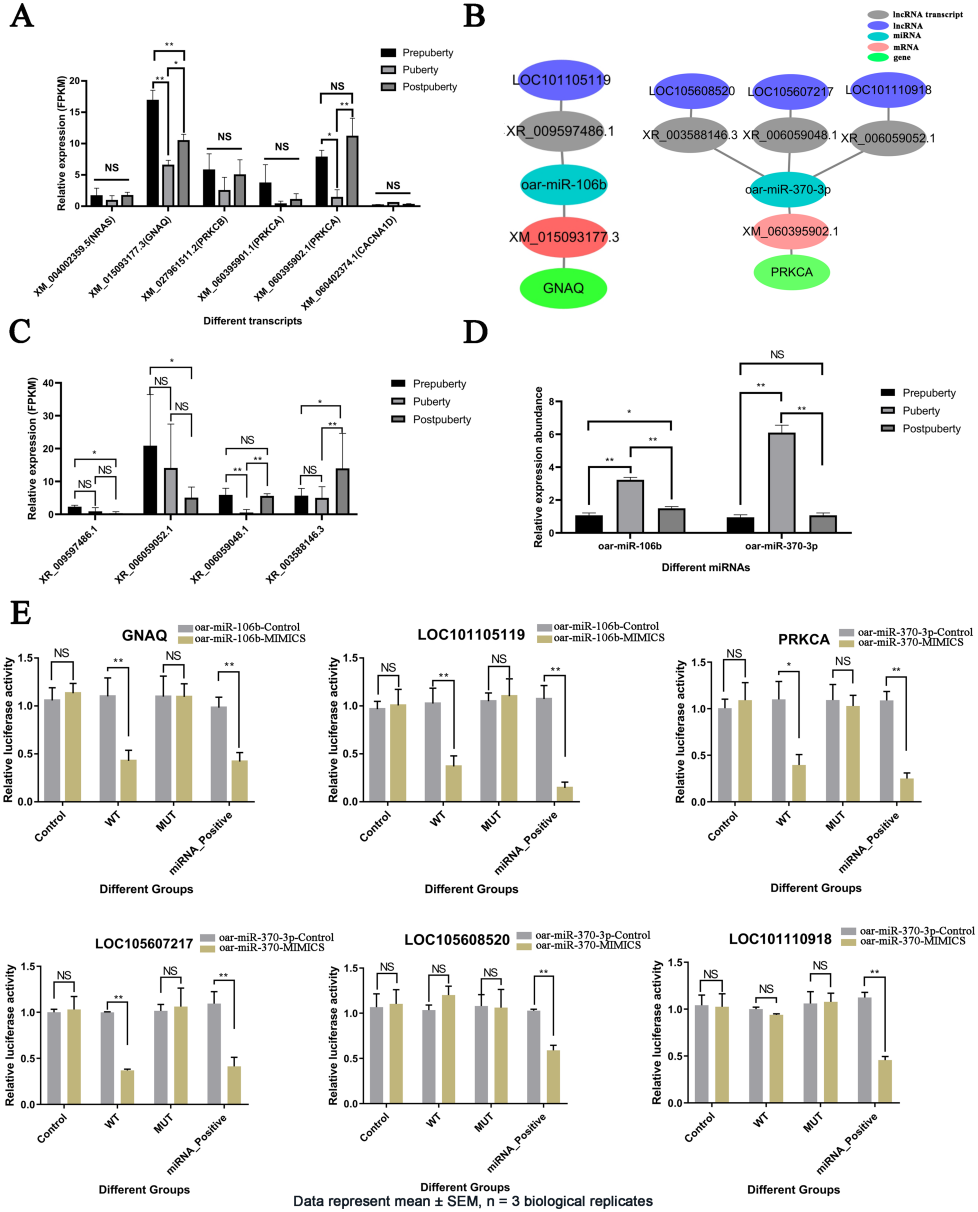
Analysis of expression patterns of hub genes in the GnRH secretion-related ceRNA network and validation of targeted interactions. **(A)** Expression (FPKM) bar plot of hub genes in the GnRH secretion-related ceRNA network. **(B)** The ceRNA relationship diagram for hub genes *GNAQ* and *PRKCA*. **(C)** Expression (FPKM) bar plot of lncRNAs in the GnRH secretion-related ceRNA network. **(D)** Expression bar plot of oar-miR-106b and oar-miR-370-3p detected by RT-qPCR. **(E)** Detection of targeting relationship of LOC101105119-oar-miR-106b-*GNAQ*, LOC105608520/LOC105607217/LOC101110918-oar-miR-370-3p-*PRKCA* using dual luciferase assay. Data are presented as mean ± SEM (*n* = 3 biological replicates). *p value <0.05, **p value <0.01, and NS, Not Significant.

Among the five hub genes of the GnRH secretion ceRNA network, *GNAQ* (XM_015093177.3) and *PRKCA* (XM_060395902.1) were differentially expressed in the three stages and were significantly lower in puberty than in prepuberty and postpuberty (FDR < 0.05) (Figure 6A). Therefore, the LOC101105119-oar-miR-106b-*GNAQ* and LOC105608520/LOC105607217/LOC101110918-oar-miR-370-3p-*PRKCA* relationships were screened for targeted relationship exploration (Figure 6B and Supplementary Figure S10). Sequencing results showed that LOC101105119 (XR_009597486.1) and LOC101110918 (XR_006059052.1) were highly expressed in prepuberty (Figure 6C). LOC105608520 (XR_003588146.3) was highly expressed in postpuberty. LOC105607217 (XR _006059048.1) was lowly expressed in puberty. RT-qPCR results showed that oar-miR-106b and oar-miR-370-3p were highly expressed in puberty (Figure 6D). The results of the dual luciferase assay showed that compared with the control group, after transfection with oar-miR-106b mimics, the relative expression of firefly luciferase in wild-type vectors and positive vectors of *GNAQ* and LOC101105119 was significantly downregulated (*p* < 0.01), and the expression of mutant vectors did not change significantly (Figure 6E). After transfection with oar-miR-370-3p mimics, the relative expression of firefly luciferase in wild-type vectors and positive vectors of *PRKCA* and LOC105607217 was significantly downregulated (*p* < 0.01), and the expression of mutant vectors did not change significantly. After transfection with oar-miR-370-3p mimics, the firefly luciferase in wild-type vectors and mutant vectors of LOC105608520 and LOC101110918 did not change significantly, and the positive vector was significantly downregulated (*p* < 0.01). Dual luciferase assays showed that oar-miR-106b had targeted regulatory relationships with *GNAQ* and LOC101105119, while oar-miR-370-3p had targeted regulatory relationships with *PRKCA* and LOC105607217, but had no targeted regulatory relationship with LOC105608520 and LOC101110918.

**Figure 7:**
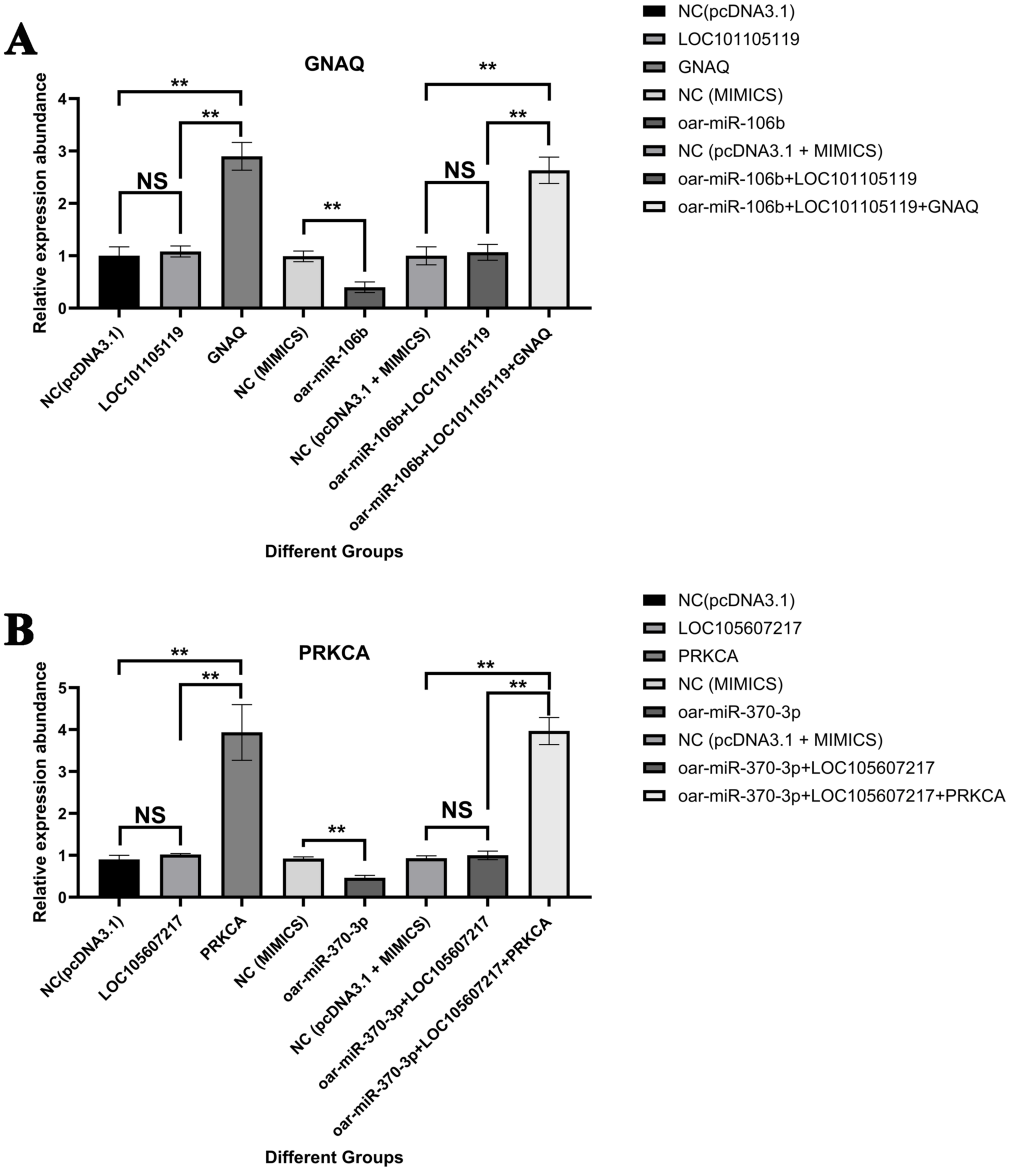
Validation results of LOC101105119-oar-miR-106b-*GNAQ* and LOC105607217-oar-miR-370-3p-*PRKCA* ceRNA regulatory relationship. **(A)** Validation results of LOC101105119-oar-miR-106b-*GNAQ* ceRNA regulatory relationship. **(B)** Validation results of LOC105607217-oar-miR-370-3p-*PRKCA* ceRNA regulatory relationship. The bar graph shows the relative expression of *GNAQ* and *PRKCA* detected by RT-qPCR 48 h after cell transfection. The grouping information is as follows: empty pcDNA3.1 vector without sequence insertion (NC(pcDNA3.1)); gene overexpression vector (gene); lncRNA overexpression vector (lncRNA); miRNA control group (NC (MIMICS)); miRNA overexpression group (miRNA); NC (pcDNA3.1 + MIMICS); miRNA and lncRNA co-overexpression group (miRNA + lncRNA); miRNA, lncRNA, and gene co-overexpression group (miRNA + lncRNA + gene). *p value <0.05, **p value < 0.01, and NS, Not Significant.

Cell transfection revealed that the transfection efficiency of the overexpression vector was above 64% (Supplementary Figure S11). The results of the cell transfection experiment showed that compared with the control group (Figure 7A), the LOC101105119 overexpression group had no significant effect on the expression of *GNAQ*, while the *GNAQ* overexpression group had a significant upregulation of *GNAQ* expression (*p* < 0.01). Overexpression of oar-miR-106b significantly downregulated the expression of *GNAQ* (*p* < 0.01), while oar-miR-106b + LOC101105119 co-transfected cells did not significantly change the expression of *GNAQ* compared with the control group. When oar-miR-106b + LOC101105119 + *GNAQ* were co-transfected into cells, *GNAQ* was significantly upregulated compared with the control group (*p* < 0.01). Similarly, compared with the control group, overexpression of LOC105607217 alone had no significant effect on *PRKCA* expression, while overexpression of oar-miR-370-3p significantly downregulated *PRKCA* expression (Figure 7B). Compared with the control group, the expression of *PRKCA* in the oar-miR-370-3p + LOC105607217 group had no significant change, while the expression of *PRKCA* in the oar-miR-370-3p + LOC105607217 + *PRKCA* group was significantly upregulated (*p* < 0.01).

### 3.8 Analysis of differentially expressed lncRNA expression patterns and co-expression analysis with mRNA

**Figure 8:**
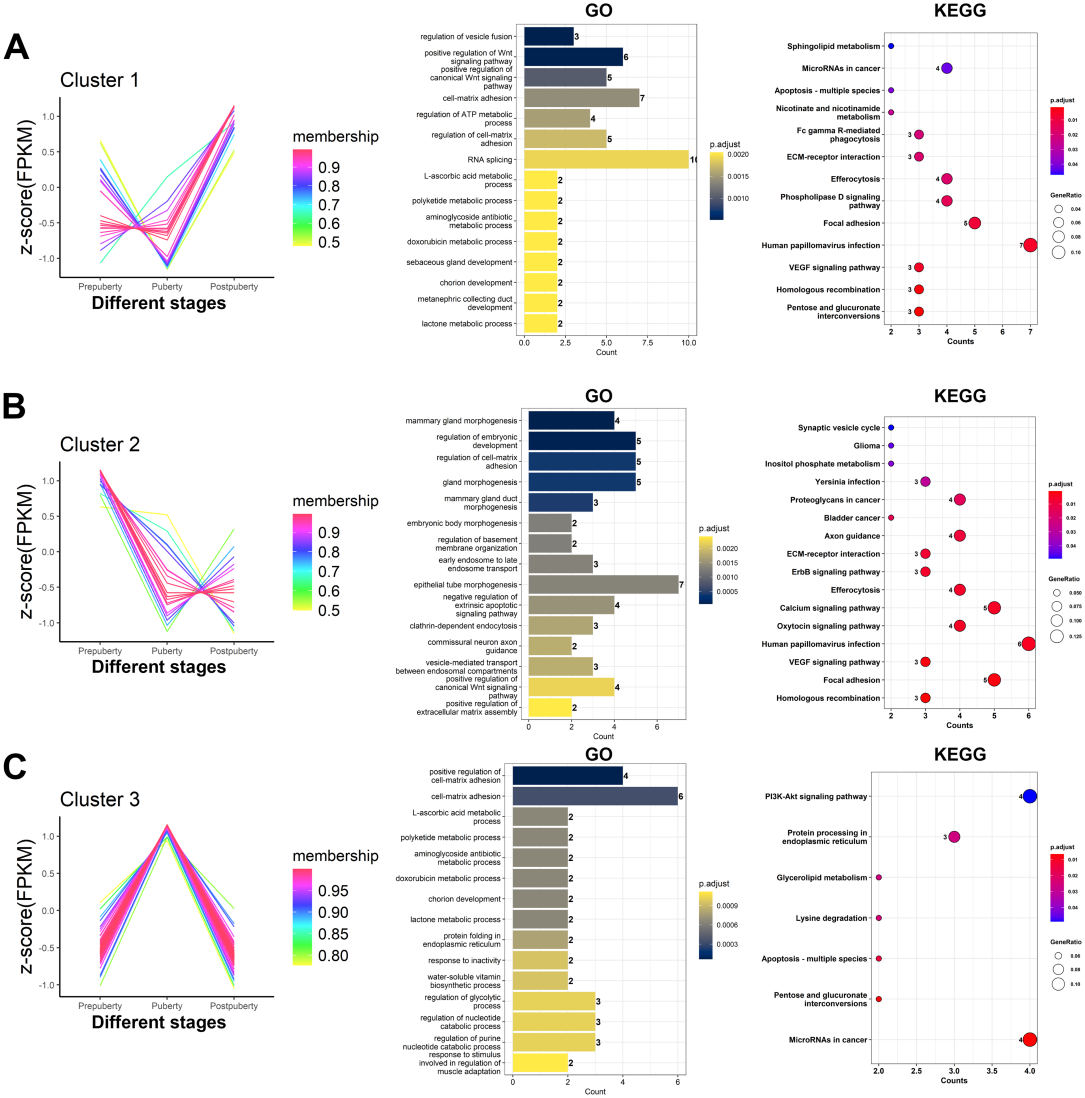
Expression patterns and target genes function analysis of differentially expressed lncRNAs. The 129 differentially expressed lncRNAs were grouped based on their expression levels, resulting in the identification of three distinct clusters of lncRNAs. **(A–C)** cluster1, cluster2, and cluster3 lncRNAs, respectively. According to the lncRNA-mRNA co-expression relationship, the target genes corresponding to the lncRNAs in each cluster were obtained. The bar chart and dotplot show the GO annotation and KEGG pathway enrichment analysis results of the target genes corresponding to lncRNA, respectively.

According to the expression of differentially expressed lncRNAs, a total of 129 lncRNAs were grouped into three clusters (Figure 8 and Supplementary Table S9). Cluster 1 comprised 27 lncRNAs, which exhibited significantly high expression levels specifically in postpuberty. Cluster 2 consisted of 23 lncRNAs, which showed specific high expression in prepubety. Cluster 3 included 79 lncRNAs, which demonstrated specific high expression during the puberty. Co-expression analysis results (Supplementary Table S10) revealed that out of the differentially expressed lncRNAs, there were a total of 1,194 co-expression relationships with 205 protein-coding mRNAs involving 58 lncRNAs. The GO and KEGG analysis of the co-expressed mRNAs in each cluster of lncRNAs (Supplementary Table S11) revealed that GO terms such as regulation of vesicle fusion, positive regulation of Wnt signaling pathway, positive regulation of canonical Wnt signaling pathway, cell-matrix adhesion, and regulation of ATP metabolic process were significantly enriched in cluster 1. In cluster 2, there was significant enrichment of GO terms related to mammary gland morphogenesis, regulation of embryonic development, regulation of cell-matrix adhesion, gland morphogenesis, mammary gland duct morphogenesis, and other aspects of mammary gland development. Additionally, signaling pathways related to the reproductive system such as the oxytocin signaling pathway, calcium signaling pathway, and ErbB signaling pathway were also significantly enriched. In cluster 3, there was a significant enrichment of GO terms related to structural support and signal transduction. These included terms such as positive regulation of cell-matrix adhesion, cell-matrix adhesion, and chorion development.

### 3.9 LncRNA regulates the oxytocin signaling pathway through a co-expression relationship

**Figure 9:**
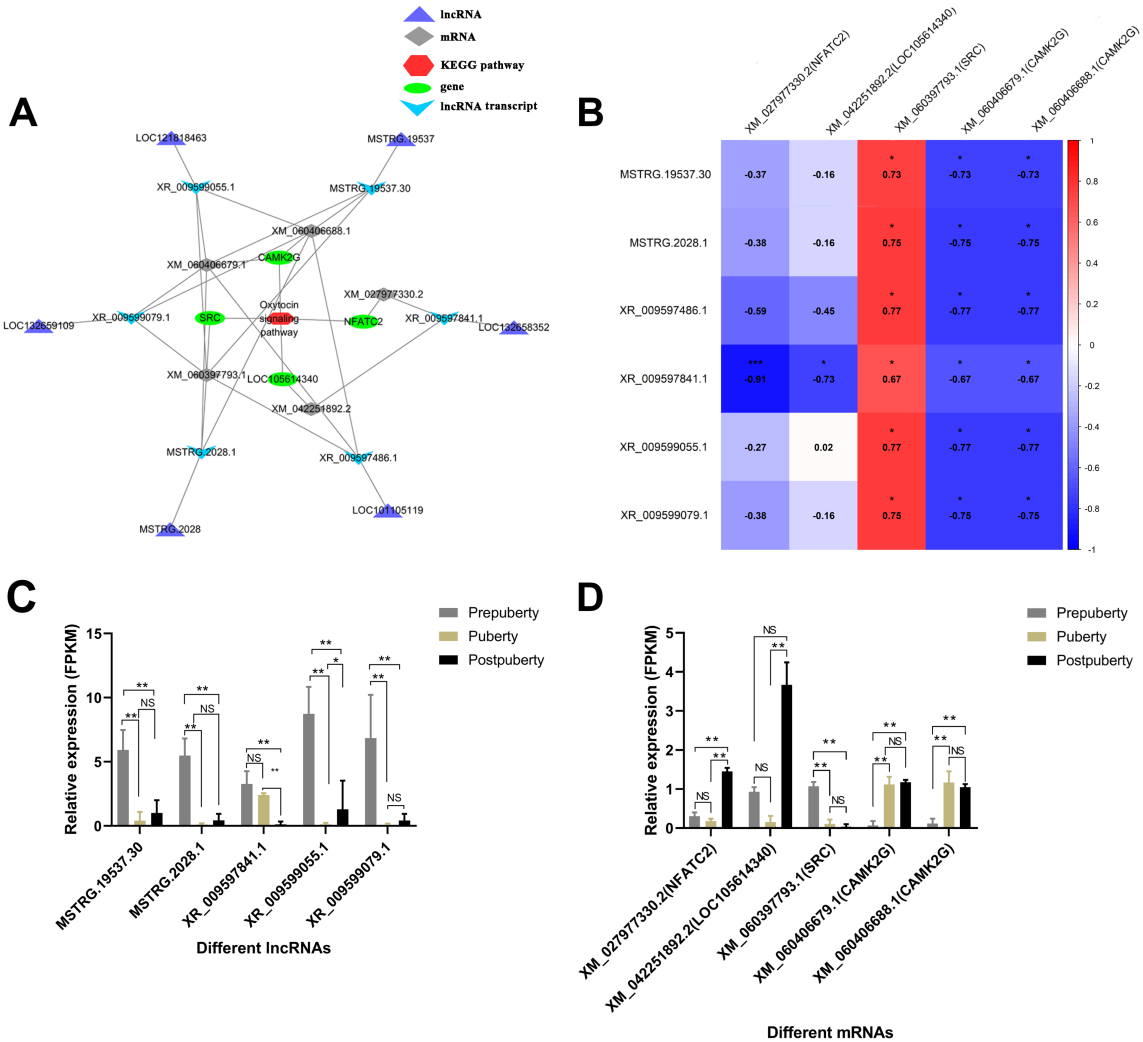
Construction of a lncRNA-mRNA co-expression network associated with the oxytocin signaling pathway. **(A)** A lncRNA-mRNA co-expression network associated with the oxytocin signaling pathway. **(B)** Heat map illustrating the correlation between lncRNA and mRNA expression levels. **(C)** Bar graph depicting the expression levels of lncRNAs in prepuberty, puberty, and postpuberty. **(D)** Bar graph depicting the expression levels of mRNAs in prepuberty, puberty, and postpuberty. *p value < 0.05, **p value < 0.01, and NS, Not Significant.

According to the above KEGG enrichment analysis results, a co-expression relationship network related to the oxytocin signaling pathway was constructed (Figure 9). The network contains 5 mRNAs derived from 4 genes (*CAMK2G*, *SRC*, LOC105614340, and *NFATC2*), and 6 lncRNA transcripts derived from 6 lncRNA genes (MSTRG.19537, LOC121818463, LOC132659109, MSTRG.2028, LOC101105119, and LOC132658352). XR_009597486.1 (LOC101105119) and XR_009599055.1 (LOC121818463) were significantly positively correlated with XM_060397793.1 (*SRC*) (*p* < 0.05) (Figure 9B). It is well established that steroid receptor coactivator (*SRC*) is a nuclear receptor coactivator that plays a crucial role in the reproductive system of mammal (45). The study revealed that SRC is involved in regulating the activity of sex hormone receptors, such as estrogen receptor (ER) and androgen receptor (AR), thereby impacting the development of germ cells, the synthesis of reproductive hormones, and the proliferation of gonads. Notably, it was found that SRC is highly expressed in prepuberty (Figure 9D), suggesting that the two co-expressed lncRNAs (LOC101105119 and LOC121818463) may play a role in regulating germ cell development and reproductive hormone synthesis to influence sexual maturity in sheep. *CAMK2G* is significantly upregulated during puberty and postpuberty. Additionally, XR 009597486.1 (LOC101105119) and XR 009599055.1 (LOC121818463) show a negative correlation with *CAMK2G* expression. As an essential signal-transduction protein kinase, *CAMK2G* plays a crucial role in various biological processes such as neurotransmission, muscle contraction, and cell proliferation (46). LOC101105119 and LOC121818463 may impact the function of the reproductive system by influencing the cell cycle, cell differentiation, or interacting with other signaling pathways through *CAMK2G*.

### 3.10 LncRNA regulates calcium signaling pathway through co-expression relationship

The calcium signaling pathway co-expression network included six mRNAs (Supplementary Figure S12A), derived from five genes (*CAMK2G*, LOC105614340, *SPHK2*, *NFATC2*, and *P2RX6*), and six lncRNA transcripts derived from six lncRNA genes (LOC132658352, MSTRG.2028, LOC121818463, MSTRG.19537, LOC101105119, and LOC132659109). P2RX6 is highly expressed during adolescence and late adolescence (Supplementary Figure S12C). LncRNAs such as XR_009597486.1 (LOC101105119), XR_009599055.1 (LOC121818463), and MSTRG.2028.1 (MSTRG.2028) are all significantly negatively correlated with *P2RX6* (Figure 9B). It is known that the *P2RX6* receptor acts as an ion channel on the cell membrane, specifically binding to and responding to adenosine triphosphate (ATP). In the egg, the function of the *P2X6* receptor may be related to the maturation of the egg and early development after fertilization (47). ATP signaling through the P2X6 receptor may play a role in embryonic development stage after fertilization. Therefore, it is suggested that these lncRNAs may play an important biological role in regulating germ cell function and reproductive system by affecting *P2RX6*. *SPHK2* is significantly upregulated in postpuberty, and XR 009597841.1 (LOC132658352) shows a negative correlation with *SPHK2* expression. Additionally, it is well-established that the activity of *SPHK2* is closely linked to cell apoptosis and survival processes in academic literature (48). In the reproductive system, maintaining a balance between cell survival and death is crucial for the quantity and quality of eggs. Additionally, *SPHK2* plays a role in regulating the metabolism of signal lipids on the cell membrane, which may impact the signal transduction pathway of sex hormones. Sex hormones are key regulatory factors in the reproductive system, playing an important role in the development, maturation, and function of eggs sperm, and gonads (49). Therefore, it is evident that LOC132658352 may influence the function of the reproductive system by regulating *SPHK2*.

## 4 Discussion

Puberty estrus is a crucial stage in the reproductive cycle of mammals. In the case of livestock such as sheep, the initiation mechanism of estrus not only impacts reproductive efficiency but also directly influences their production performance (50). In recent years, with the advancement of high-throughput sequencing technology, transcriptome sequencing (RNA-Seq) has demonstrated significant potential in uncovering the regulatory mechanism of gene expression (51, 52). Specifically, lncRNA, as a vital type of noncoding RNA, has garnered increasing attention for its role in transcriptional regulation, post-transcriptional control, and epigenetic processes (6, 53, 54). This study aims to investigate the regulatory role of lncRNA in the puberty estrus initiation mechanism through transcriptome sequencing of sheep hypothalamus tissue. The findings aim to provide a theoretical basis for efficient sheep breeding.

This study has revealed that the number of intergenic lncRNAs in the sheep hypothalamus exceeds the number of intronic lncRNAs (Figure 1D and Supplementary Table S5). This finding is consistent with previous studies conducted in sheep hypothalamus (11, 55), adipocytes (56, 57), muscle (58), and lung (59), but contradicts the results from research on the uterus (60), and pineal gland (61). These findings suggest that the origins of lncRNAs vary across different tissues and developmental stages in sheep. Furthermore, this study observed that mRNA generally contains 5–6 exons, whereas lncRNA typically has only 2–3 exons, highlighting a notable difference in exon count (Figures 1G,H). This finding is consistent with previous studies of the hypothalamus in sheep (55) and goats (62). This disparity can be attributed to the complex gene structure of mRNA, which encodes proteins and requires a greater number of exons to encode different protein regions. In contrast, lncRNA does not encode proteins and possesses a relatively simple gene structure with fewer exons (63). Additionally, the expression level of mRNA was found to be higher than that of lncRNA (Supplementary Figure S2). As mRNA serves as a template for protein synthesis, its expression level is directly influenced by the functional requirements of the organism. While lncRNA also plays crucial regulatory roles, its functions are more diverse and complex, often not necessitating high expression levels for functionality (64).

By conducting transcriptome sequencing analysis of sheep hypothalamic tissue, this study has identified 129 differentially expressed lncRNAs and 206 mRNAs (Figure 2 and Supplementary Table S12). A study conducted sequencing of the hypothalamus of monotocous and polytocous sheep during the follicular and luteal phases, identifying 622 and 809 differentially expressed lncRNAs, respectively (55). In addition, differentially expressed lncRNAs were found in comparisons of hypothalamic sequencing of adolescent goats and rats (65), hypothalamic sequencing of goats with different litters (66), and hypothalamic sequencing of goats at different developmental stages from birth to sexual maturity (62). The above not only deepens the understanding of the complexity of the sheep hypothalamic transcriptome, but also reveals the potential role of lncRNA in regulating the reproductive cycle (such as the follicular and luteal phases, different stages of puberty) and reproductive performance (such as single births and multiple births). Specifically, the study has revealed that lncRNA can regulate the expression of hypothalamus-related genes through various mechanisms. For instance, lncRNA can directly modulate the expression of adjacent genes via cis-acting elements such as enhancers, promoters, and silencers (62, 67, 68). In this study, several lncRNAs associated with hypothalamic hormone and neural regulation functions have been identified (Supplementary Table S7). These lncRNAs may exert their regulatory effects on physiological processes such as the estrogen biosynthetic process, dopamine transport, and glycolipid metabolic process through cis-acting mechanisms, thereby influencing the onset of estrus (Figure 3A). It is well-established that during puberty, the ovaries become active and estrogen levels rise rapidly, which in turn promotes the development of female reproductive organs and the emergence of secondary sexual characteristics such as breast development, pelvic widening, and subcutaneous fat deposition in preparation for the onset of estrus (i.e., sexual maturity) (69). During puberty, there are significant changes in the emotions and behaviors of sheep, which are closely related to the development and functional changes of the dopamine system. Increased levels of dopamine may enhance the exploratory desire and adventurous behavior of sheep, as well as affect their interest in and perception of attraction to the opposite sex (70). Glucose and lipid metabolism refer to the processes of synthesis, decomposition, conversion, and utilization of glucose and lipids in the body (71). Puberty is a critical period for growth and development, requiring substantial energy support. The process of glucose and lipid metabolism provides the body with necessary energy and material basis to support the development of reproductive organs and appearance of secondary sexual characteristics (72). In summary, there exists a complex and close relationship between estrogen biosynthesis, dopamine transport, glucose and lipid metabolism, as well as pubertal estrus. lncRNA can regulate these physiological processes through homeostasis, affecting the growth, development, and sexual maturation process during puberty.

The secretion of reproductive hormones plays a crucial role in the initiation of estrus (73). Certain lncRNAs in the hypothalamus may function as ceRNAs, binding to specific miRNAs and influencing the secretion of luteinizing hormone (LH) and follicle-stimulating hormone (FSH) by regulating the expression of genes related to the anterior pituitary (74). For instance, certain lncRNAs may impact the ovary’s sensitivity to FSH by modulating the expression of FSH receptors, thereby regulating follicle development and ovulation (75). In this study, several lncRNAs associated with the secretion of reproductive hormones have been identified. These lncRNAs may impact the secretion of LH and FSH by regulating the expression of genes involved in signaling pathways such as GnRH secretion, oxytocin signaling pathway, estrogen signaling pathway, and insulin secretion, thereby influencing the process of estrus initiation (Figures 5, 9). This study verified that the two groups of ceRNA relationships, oar-miR-106b + LOC101105119 + *GNAQ*, and oar-miR-370-3p + LOC105607217 + *PRKCA*, came from the GnRH secretion regulatory network (Figure 6). It is known that the activation of GnRH neurons and changes in pulse secretion patterns are key to puberty and estrus cycles. *GNAQ*, as a Gq protein subunit, is responsible for activating the phosphatidylinositol bisphosphate (PIP2) pathway through the GnRH receptor to produce inositol trisphosphate (IP3) and diacylglycerol (DAG), signaling molecules that increase intracellular calcium concentrations and activate PKC (76). PRKCA (PKCα) is a subtype of PKC. After being activated by DAG, it further regulates the activity of GnRH neurons and enhances the frequency and amplitude of GnRH secretion (77). In this study, it was found that both *GNAQ* and *PRKCA* were highly expressed before puberty (Figure 6A). Prepubertal peak expression of *GNAQ*/*PRKCA* suggests their role in priming the HPG axis for estrus initiation, possibly by sensitizing GnRH neurons to external stimuli (78). Therefore, the high expression of *GNAQ* and *PRKCA* before puberty may be a key mechanism for their role in initiating puberty and regulating the estrous cycle. Furthermore, there was a significant enrichment of signaling pathways related to signal transduction, including the Rap1 signaling pathway, calcium signaling pathway, and MAPK signaling pathway (Figure 9 and Supplementary Figure S12). Pubertal estrus involves the development and hormonal regulation of the reproductive system; cell-to-cell signaling and cell adhesion are likely to play crucial roles in this process. The Rap1 signaling pathway may indirectly influence puberty estrus by regulating processes such as adhesion, migration, and differentiation of germ cells (79). During puberty, the secretion and regulation of hormones may be accompanied by changes in intracellular calcium ions. Calcium signaling pathways may participate in the regulation of puberty estrus by controlling hormone receptor activity, intracellular signal transduction, and gene expression (80). The MAPK signaling pathways play a crucial role in the development and hormonal regulation of the reproductive system. They may participate in regulating processes such as germ cell proliferation, differentiation, apoptosis, as well as affecting hormone secretion and sensitivity during puberty estrus (81). In conclusion, this study has found that certain lncRNAs in the hypothalamus may function as ceRNAs by binding to specific miRNAs, thereby influencing the expression of genes related to estrus initiation and subsequently impacting the estrus process.

There is a significant correlation between the expression levels of lncRNA and mRNA (82). A study found 14 pairs of differentially expressed lncRNA-mRNA interactions in the hypothalamus of high- and low-fertility goats, some of which directly or indirectly reflect the relationship between the hypothalamus and goat fertility (12). This study has revealed that this relationship may indirectly regulate mRNA expression by impacting cell-matrix adhesion, material metabolism pathways, hormone signaling pathways, and reproductive organ development (Figure 8). These findings suggest that lncRNA plays a crucial role in fine-tuning the regulation of puberty estrus. By regulating the expression of genes related to extracellular matrix interaction, lncRNA may influence the adhesion and migration ability of cells, which are essential processes for tissue development and organ function. Furthermore, it indirectly regulates the morphological changes of reproductive tissues associated with estrus (83). Puberty estrus is characterized by significant changes in hormone levels and energy metabolism within the body. The study suggests that lncRNA may impact germ cell development and maturation by regulating the expression of energy metabolism-related mRNA such as fatty acid synthesis/decomposition. Additionally, sex hormones like estrogen and progesterone play a pivotal role in puberty estrus (84), and lncRNA can participate in their regulation through influencing synthesis, transport, receptor expression, and signal transduction to finely tune the estrus cycle. Moreover, lncRNA also influences the structure and function of reproductive organs (such as ovaries and uterus) by affecting gene expression related to their development. This provides a basis for smooth reproductive activities during estrus. In conclusion, this study highlights how lncRNA’s regulatory role in co-expression with mRNA enables organisms to adapt their estrus cycles according to internal and external environmental changes for survival and reproduction needs. This fine-tuned regulation holds great significance for sheep population reproduction and evolution.

This study has several limitations and technical considerations that warrant further discussion. First, because many lncRNAs do not possess polyadenine tails, the RNA purification method based on poly-A tails used in this study may have failed to capture some lncRNA without polyadenine tails, potentially affecting their representation in the transcriptome data. Additionally, transcriptome sequencing can only detect RNAs that are actively transcribed and cannot directly reflect protein expression. Therefore, while transcriptome data provide information on gene expression at the RNA level, they do not reveal the functional activity of genes at the protein level. Another limitation is that only three samples were used per time point, which may be insufficient and affect the statistical robustness of the results. To obtain more reliable conclusions, it is generally recommended to use at least five replicates per group in transcriptomic studies (85). Moreover, transcriptome sequencing results can be influenced by factors such as sample processing, sequencing depth, and data analysis methods (86–88), which may introduce variability and impact the stability and reproducibility of the data. The transcriptome data used in this study may lead to incomplete prediction of some lncRNA structures, especially for low-expressed or low-abundance lncRNAs, whose full-length transcripts may not be fully captured. To address these limitations, we validated the expression of certain lncRNAs and their ceRNA interactions using RT-qPCR and dual-luciferase reporter assays, thereby enhancing the reliability of the results. However, to more comprehensively elucidate the regulatory role of lncRNAs in estrus initiation, future studies should integrate multi-omics technologies, such as proteomics and metabolomics, to explore the interactions between lncRNAs and other biomolecules, and their functional roles in biological processes. In addition, single-cell sequencing should be employed to resolve the spatiotemporal specificity of lncRNA expression, and ovine primary hypothalamic cells should be used for functional validation.

## 5 Conclusion

In summary, this study provides evidence that lncRNAs may contribute to the regulation of puberty-associated estrus initiation in Dolang sheep, potentially through effects on hypothalamic processes related to neuronal development and reproductive hormone regulation. Derived from transcriptome profiling and bioinformatic prediction, these results offer preliminary insights into the molecular networks underlying reproductive maturation in Dolang sheep and establish a foundation for subsequent experimental validation and potential application in sheep breeding and reproductive management.

## Data Availability

The datasets generated for this study can be found in the NCBI GEO database (https://www.ncbi.nlm.nih.gov/geo/), GEO accession number: GSE273981.

## Footnotes

^1^ https://string-db.org/

^2^ https://www.r-project.org/
