# Supplementary material for: Transcriptome sequencing of sheep hypothalamic tissue reveals the regulatory role of lncRNA in the mechanism of pubertal estrus initiation
Source: Front Vet Sci. 2025 Sep 2;12:1594040. doi: 10.3389/fvets.2025.1594040 (PMC12439674; doi:10.3389/fvets.2025.1594040)
Supplement: Supplementary file 1 [file Data_Sheet_1.zip › supplementary materials/Supplementary_Figures.docx]

Supplementary Material

# Supplementary Figures


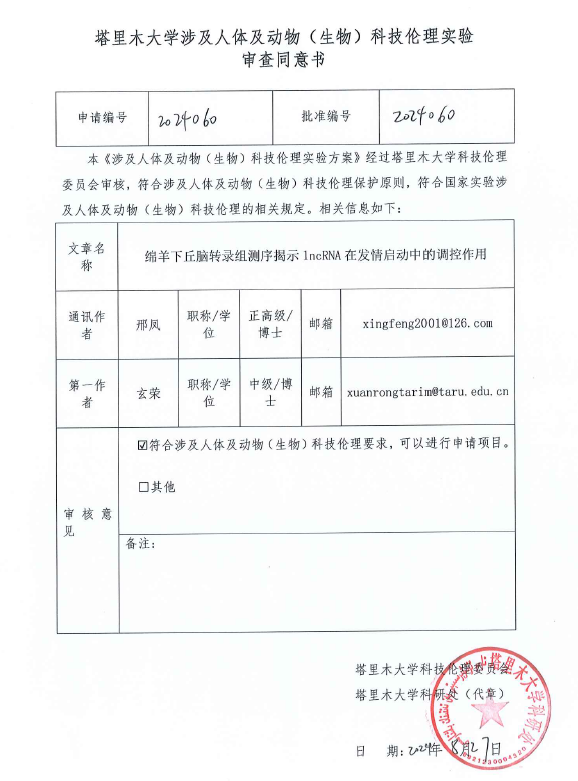


**Supplementary Figure 1.** Statement of animal ethics and approval for animal use at Tarim University.


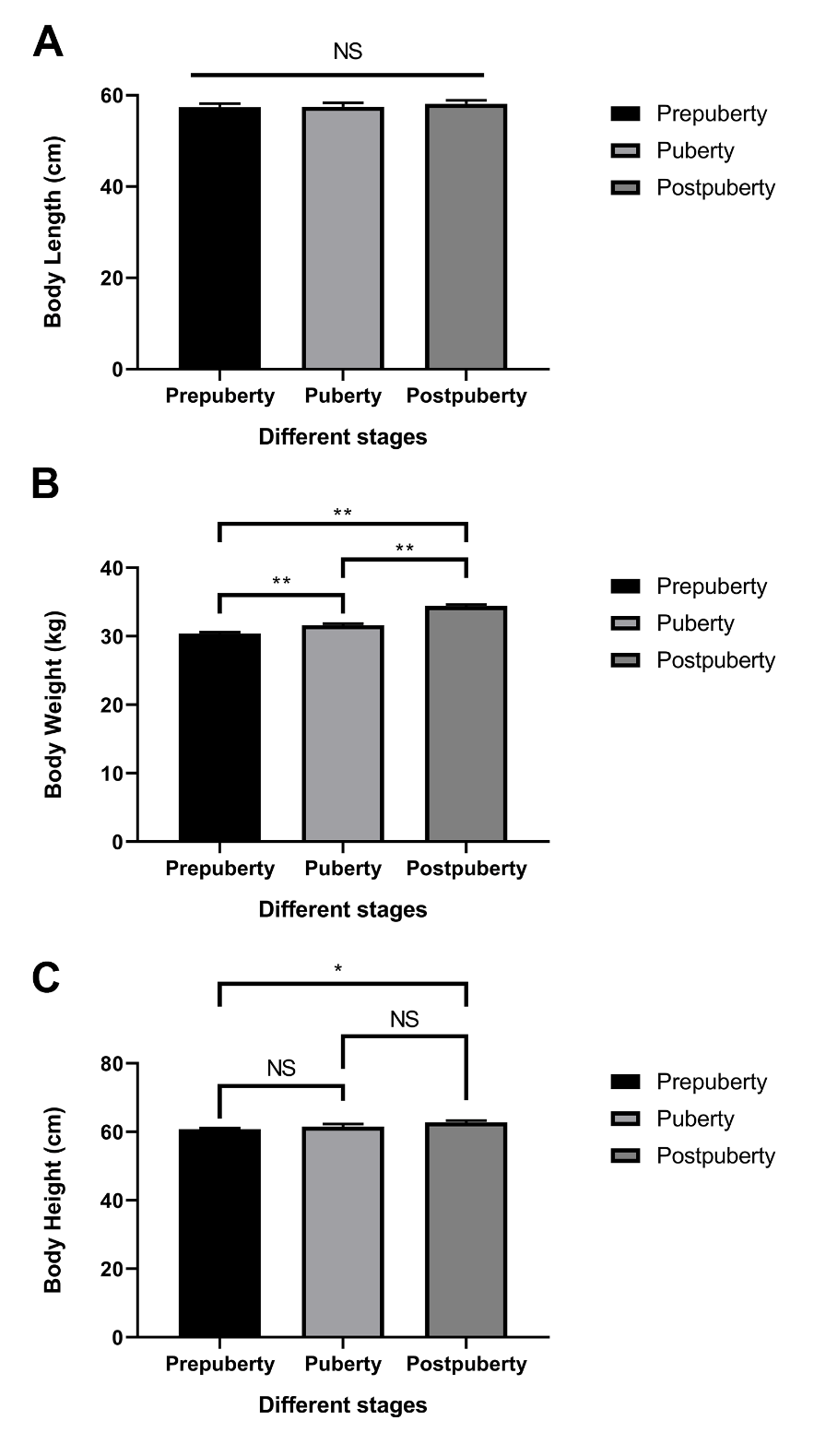


**Supplementary Figure 2.** Bar charts of body size and weight of Dolang sheep in three periods. (**A**) Comparison of body length in prepubertal, pubertal, and postpubertal sheep. (**B**) Comparison of body weights in prepubertal, pubertal, and postpubertal sheep. (**C**) Comparison of body height in prepubertal, pubertal, and postpubertal sheep. * indicates *P* value < 0.05, ** indicates *P* value < 0.01, and NS indicates Not Significant.


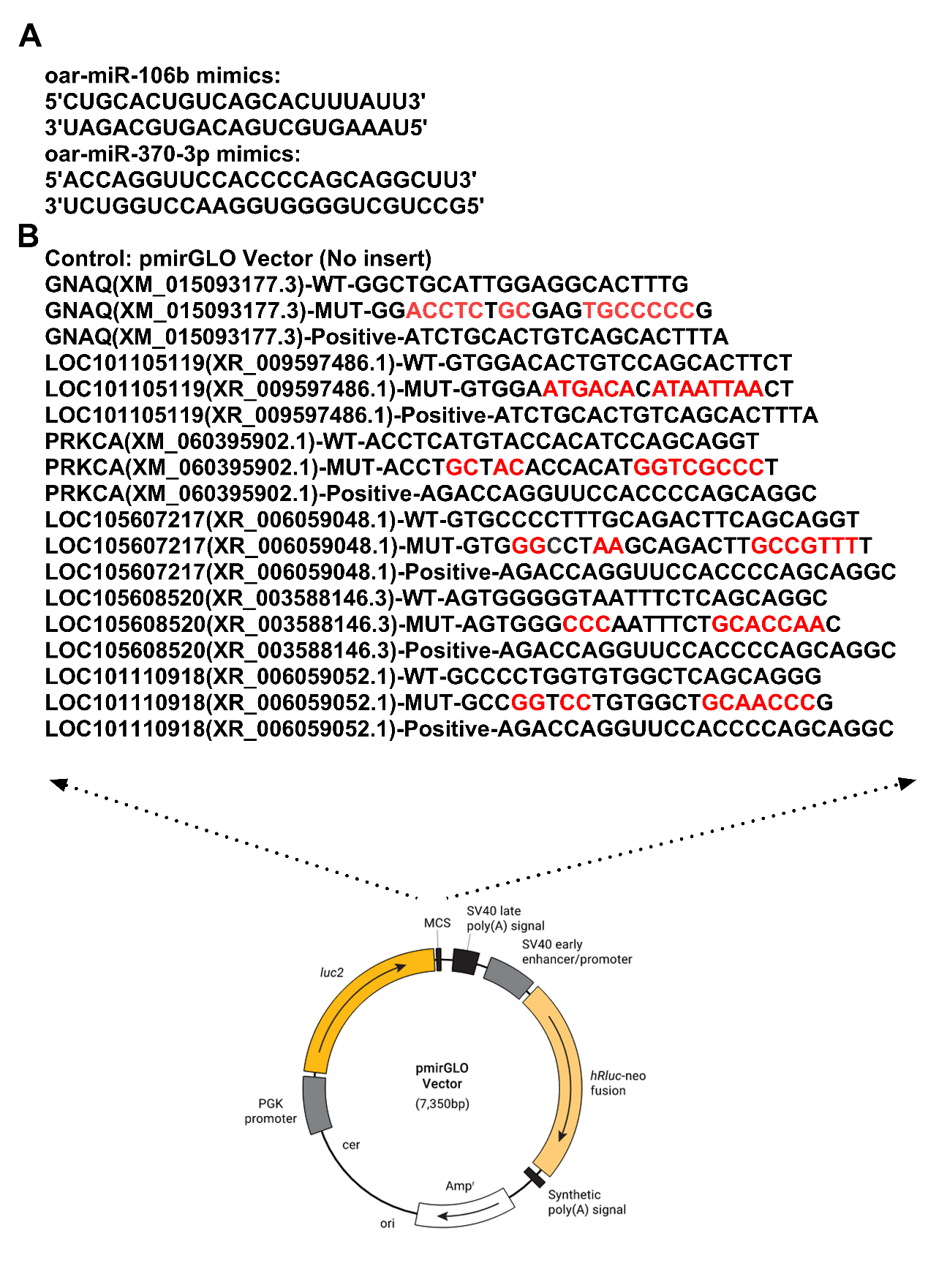


**Supplementary Figure 3.** The mimic sequences of the two miRNAs and the sequences of the wild-type, mutant, and positive vectors of *GNAQ*, LOC101105119, *PRKCA*, LOC105607217, LOC105608520, and LOC101110918. (**A**) The mimic sequences of the oar-miR-106b and oar-miR-370-3p. (**B**) The sequences of the wild-type, mutant, and positive vectors of *GNAQ*, LOC101105119, *PRKCA*, LOC105607217, LOC105608520, and LOC101110918.


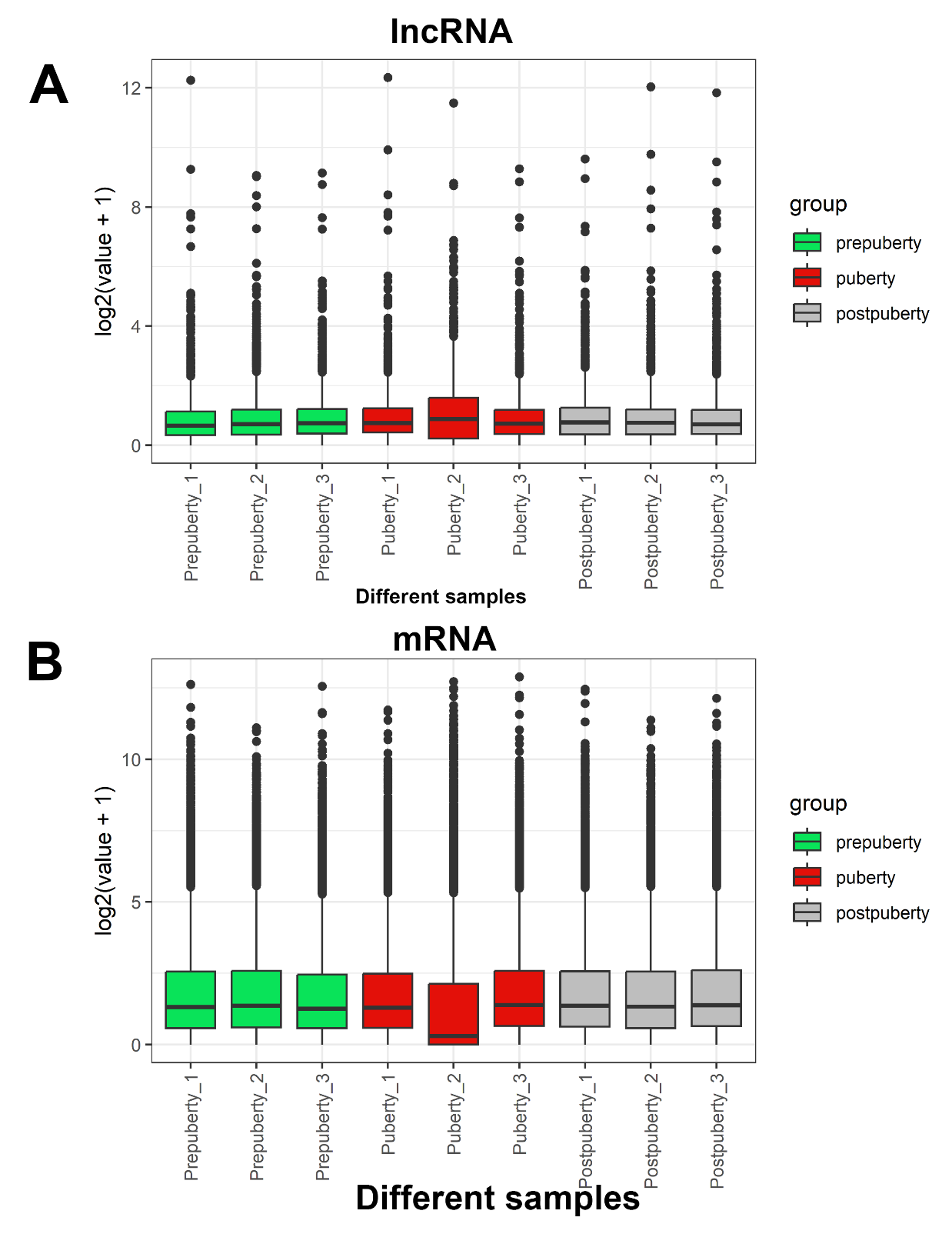


**Supplementary Figure 4.** Bar graph depicting the overall expression levels of lncRNA and mRNA in each hypothalamic sample. (**A**) The expression of lncRNA. (**B**) The expression of mRNA.


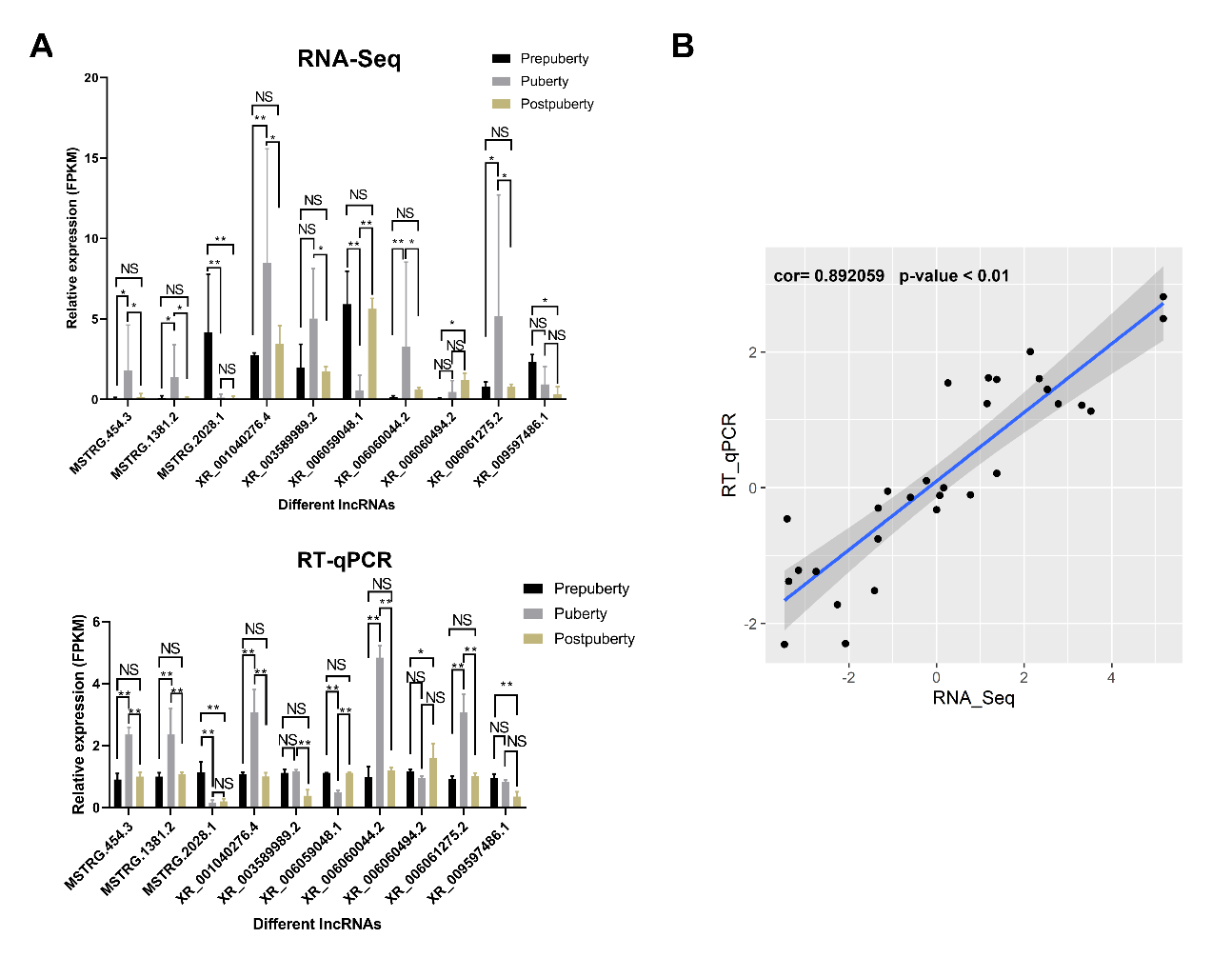


**Supplementary Figure 5.** Verification of the expression levels of 10 lncRNAs using RTqPCR. (**A**) Transcriptome sequencing to detect the expression of 10 lncRNAs. (**B**) RTqPCR to detect the expression of 10 lncRNAs. (**C**) Log2 fold change correlation between RNA-Seq and RTqPCR. * indicates *P* value < 0.05, ** indicates *P* value < 0.01, and NS indicates Not Significant.


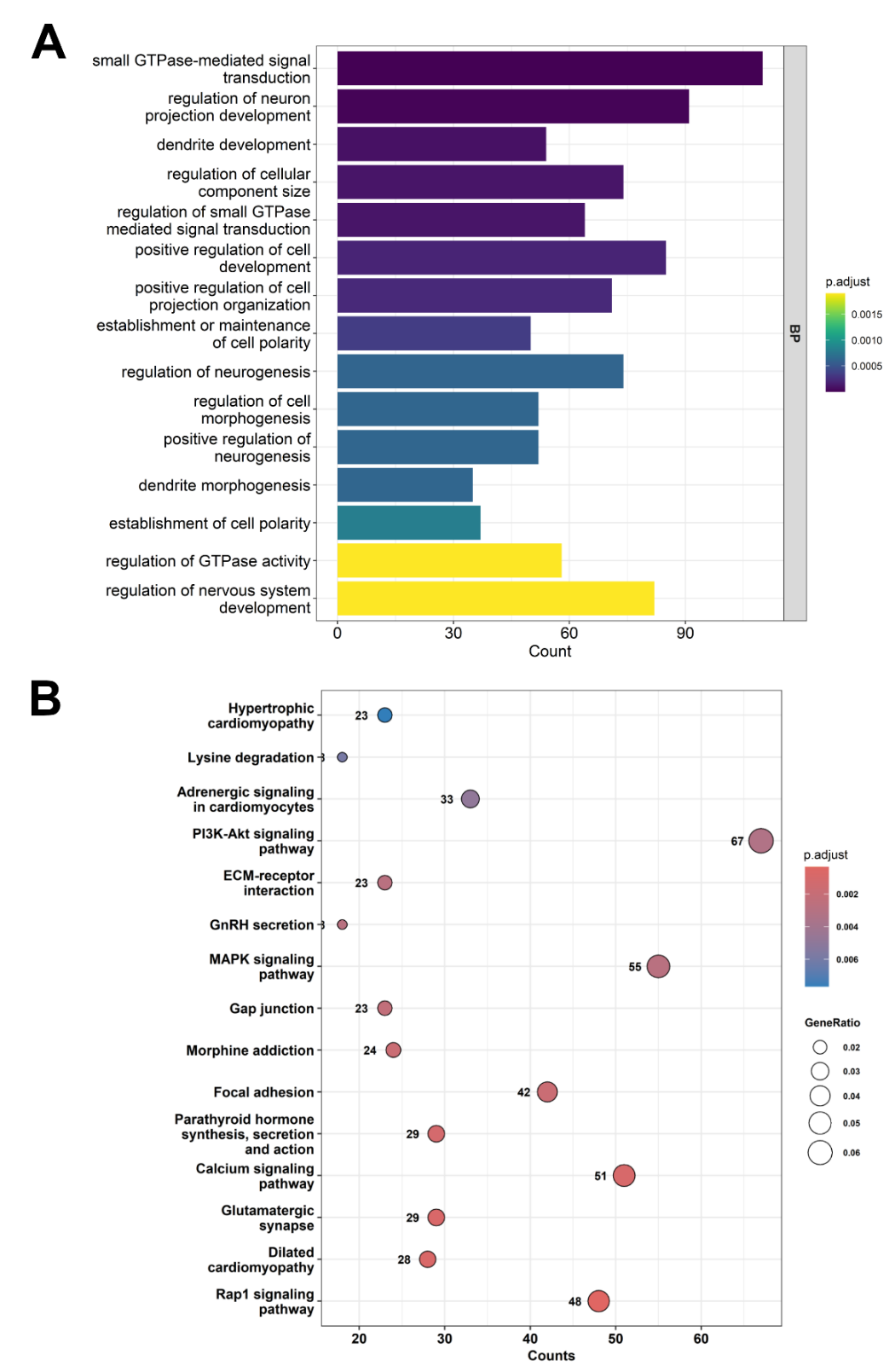


**Supplementary Figure 6.** GO functional annotation and KEGG pathway enrichment analysis of lncRNA-miRNA-mRNA ceRNA target genes. (**A**) Bar chart illustrating the GO functional annotation of lncRNA target genes in ceRNA relationships. (**B**) Dotplot illustrating the KEGG pathway enrichment analysis of lncRNA target genes in ceRNA relationships.


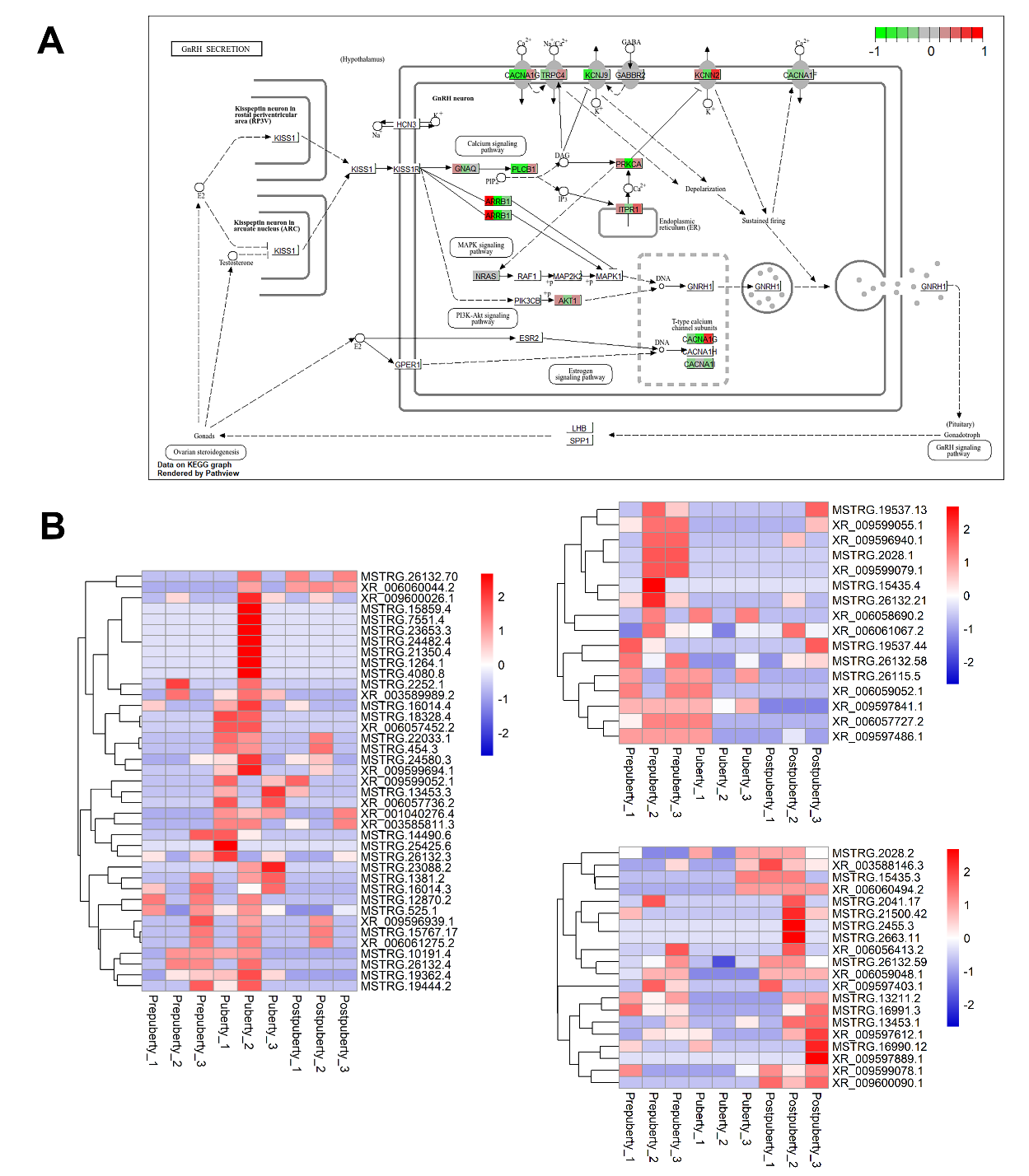


**Supplementary Figure 7.** Expression levels of mRNA and lncRNA in the ceRNA network related to the GnRH secretion signaling pathway. (**A**) Expression levels of mRNAs in the ceRNA network related to the GnRH secretion signaling pathway. (**B**) Expression heat map of lncRNAs in the ceRNA network associated with the GnRH secretion signaling pathway.


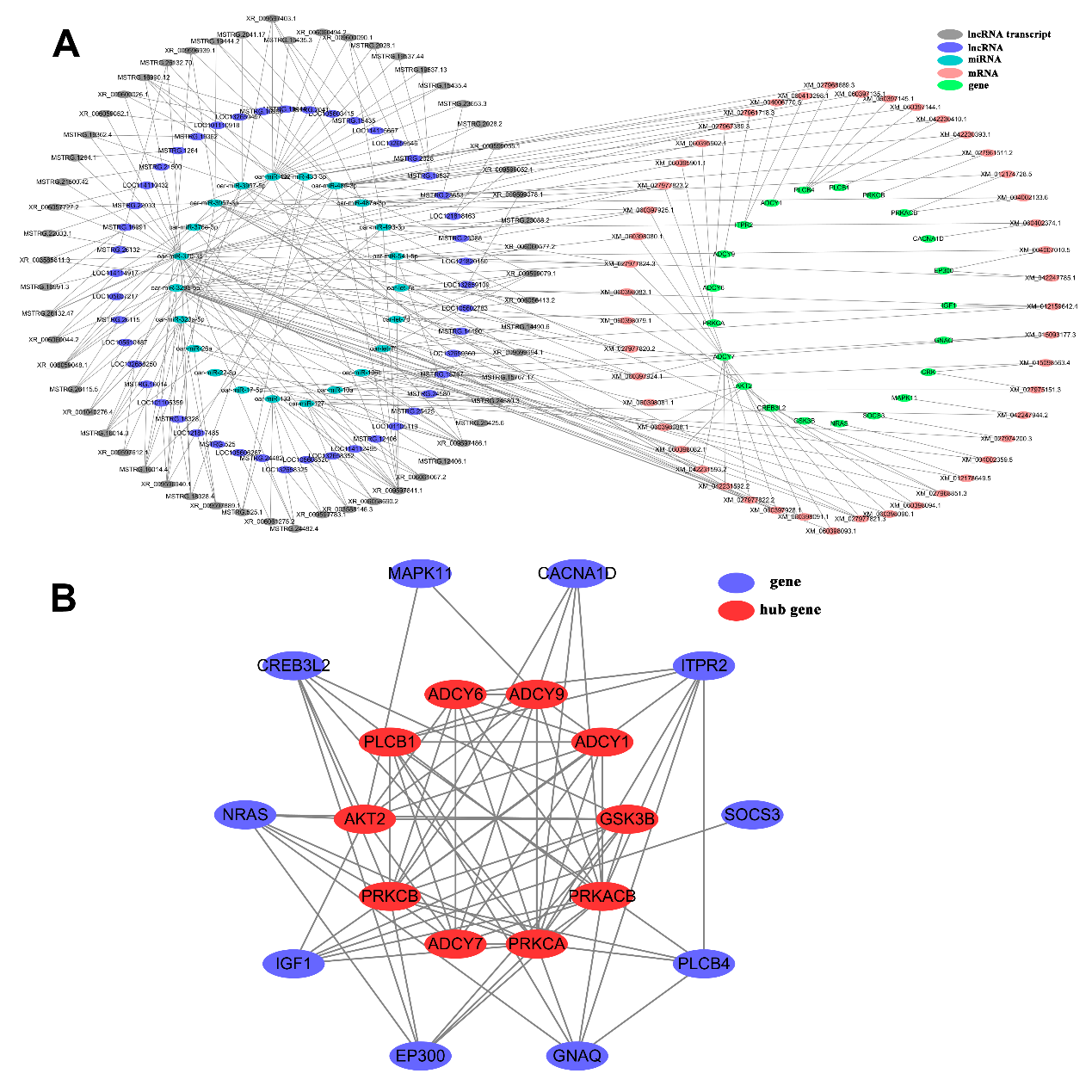


**Supplementary Figure 8.** Construction of a lncRNA-mRNA co-expression network associated with the growth hormone synthesis, secretion, and action. (**A**) A ceRNA network for growth hormone synthesis, secretion, and action. (**B**) Protein and protein interaction network diagram of genes enriched in the signaling pathway for the growth hormone synthesis, secretion, and action.


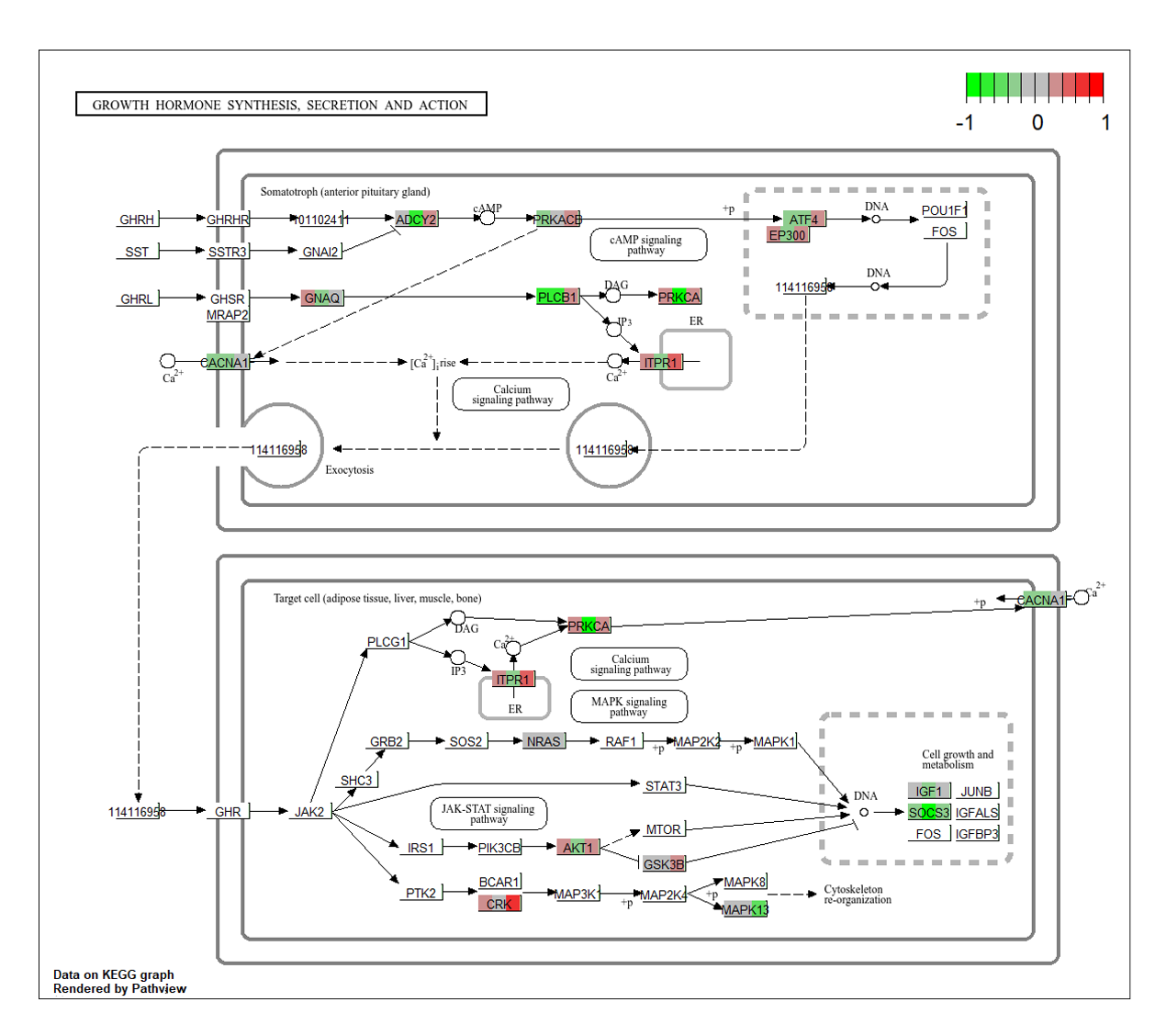


**Supplementary Figure 9.** KEGG signaling pathway diagram showing the gene expression levels related to the growth hormone synthesis, secretion, and action.


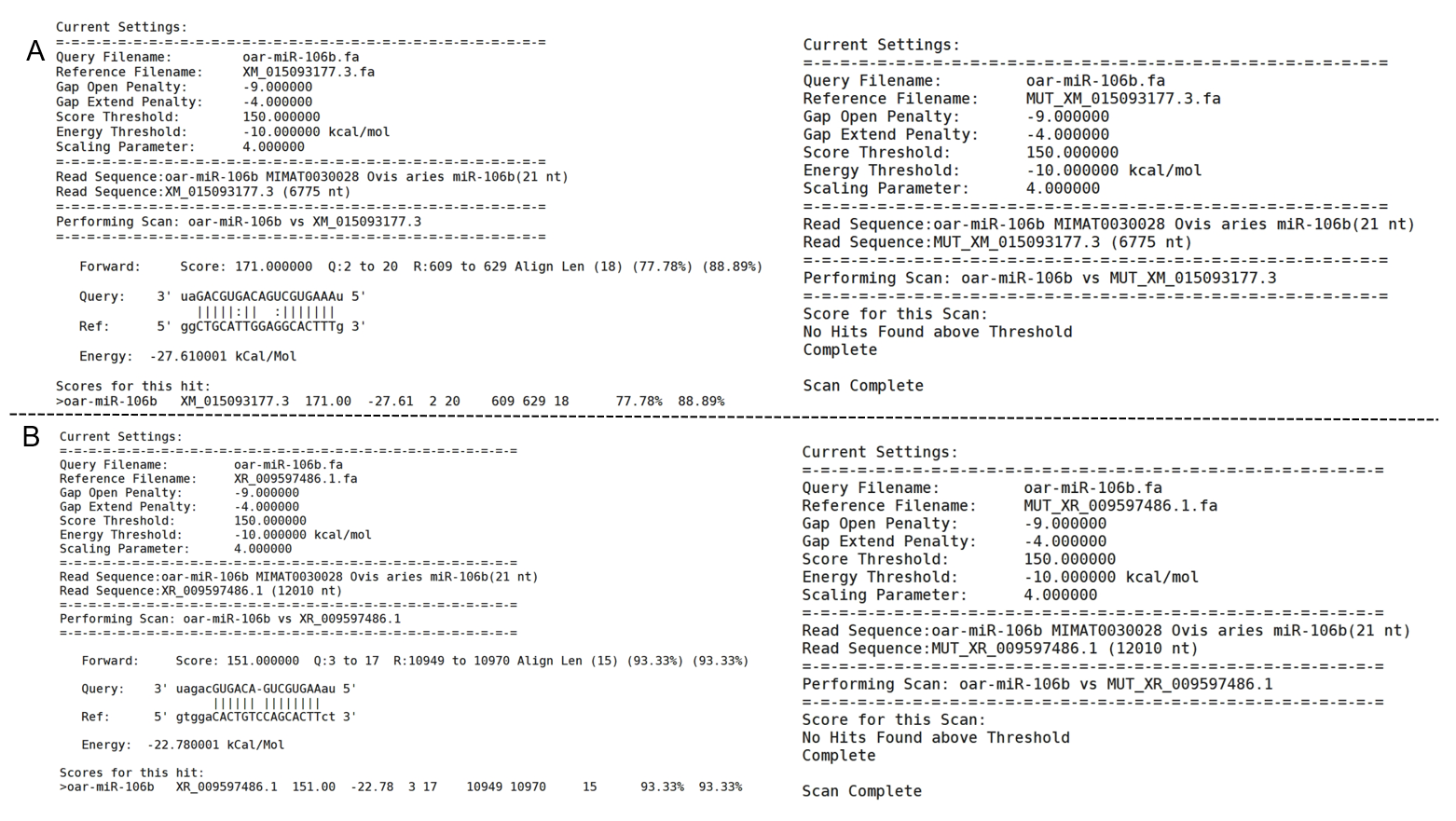

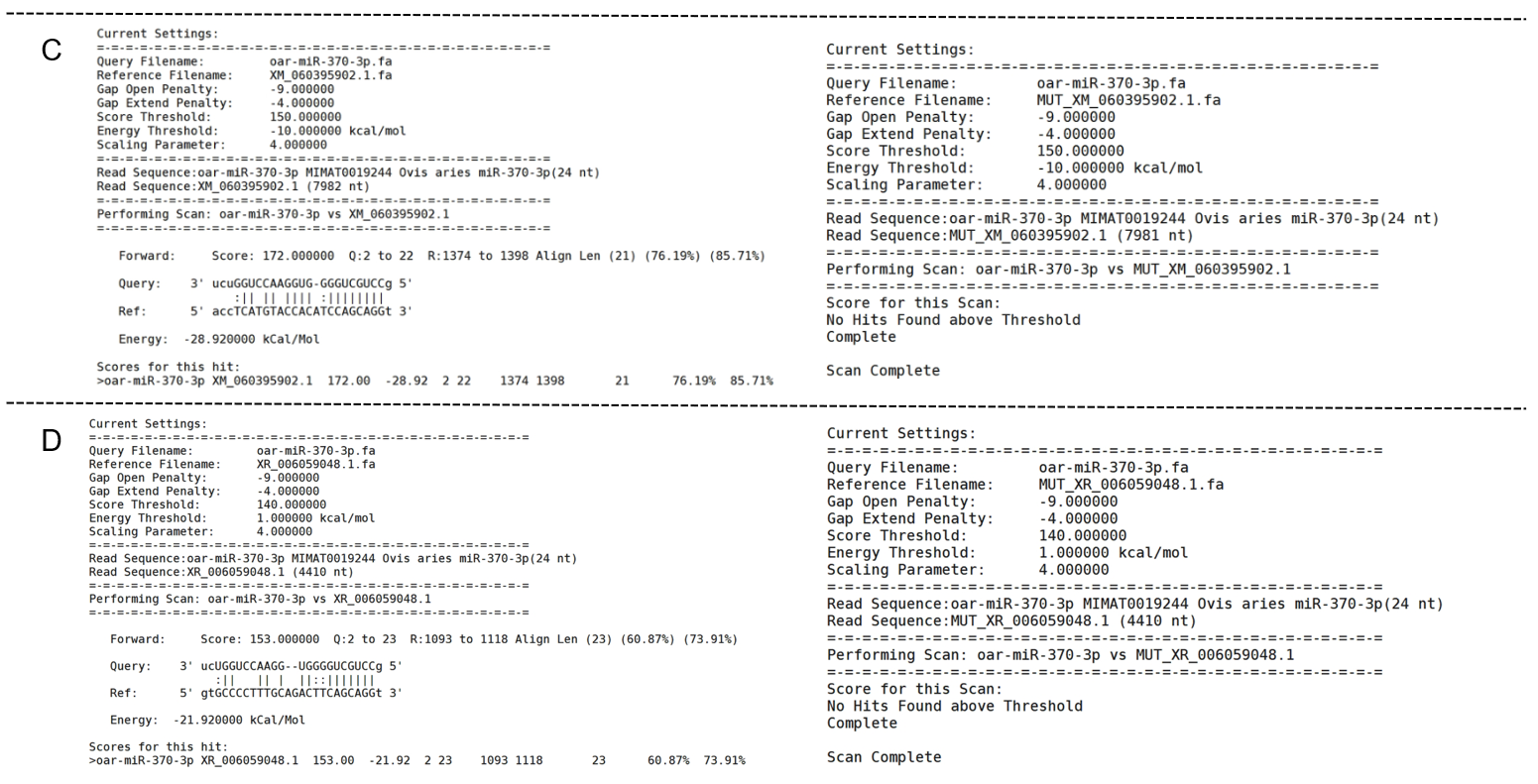


**
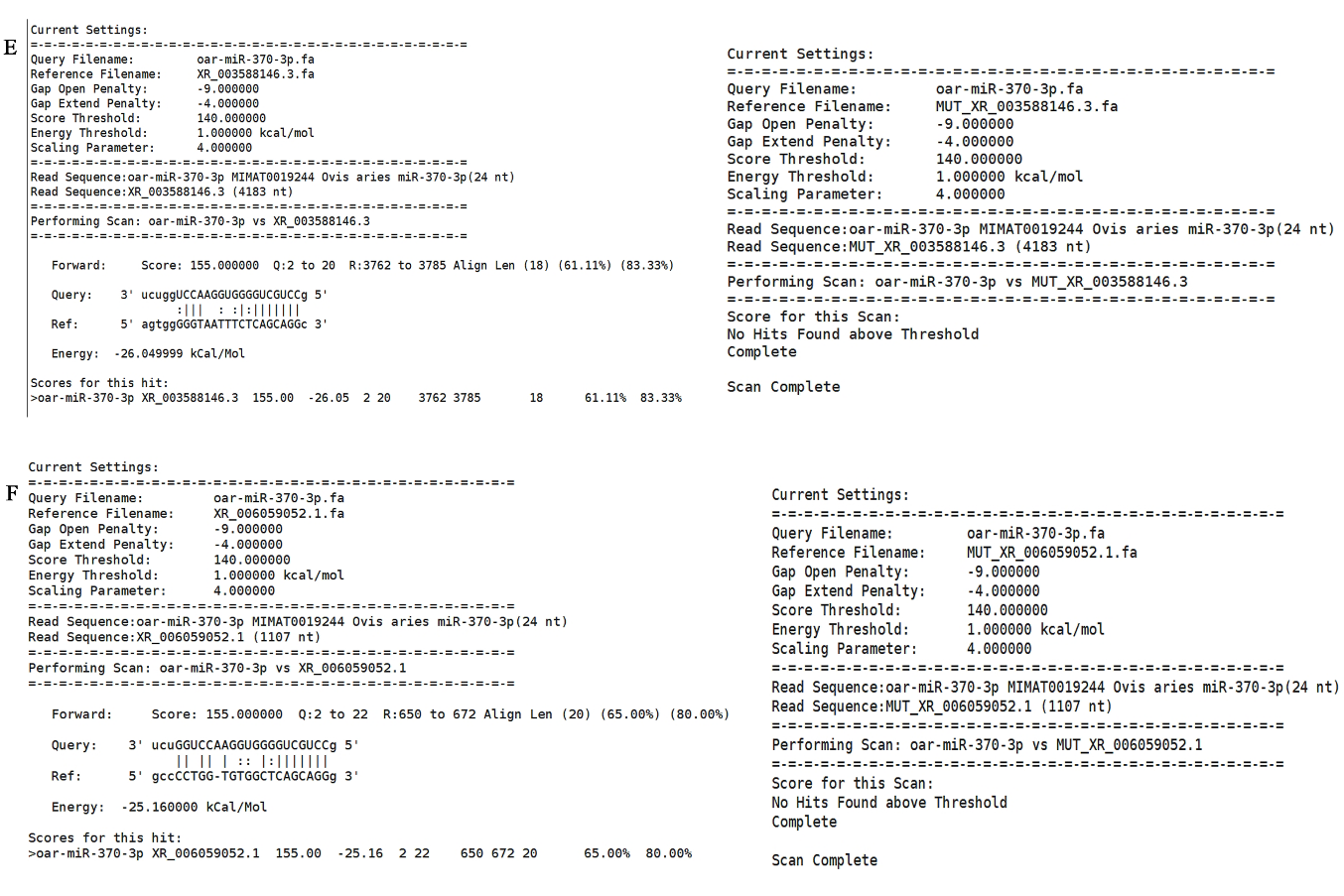
**

**Supplementary Figure 10.** Prediction of the targeting relationship of LOC101105119-oar-miR-106b-*GNAQ*、LOC105608520 / LOC105607217 / LOC101110918-oar-miR-370-3p-*PRKCA* using the miRanda software. (**A**) Prediction of the targeting relationship between oar-miR-106b and *GNAQ* / mutant *GNAQ*. (**B**) Prediction of the targeting relationship between oar-miR-106b and LOC101105119 / mutant LOC101105119. (**C**) Prediction of the targeting relationship between oar-miR-370-3p and *PRKCA*/ mutant *PRKCA*. (**D**) Prediction of the targeting relationship between oar-miR-370-3p and LOC105607217 / mutant LOC105607217. (**E**) Prediction of the targeting relationship between oar-miR-370-3p and LOC105608520 / mutant LOC105608520. (**F**) Prediction of the targeting relationship between oar-miR-370-3p and LOC101110918 / mutant LOC101110918.


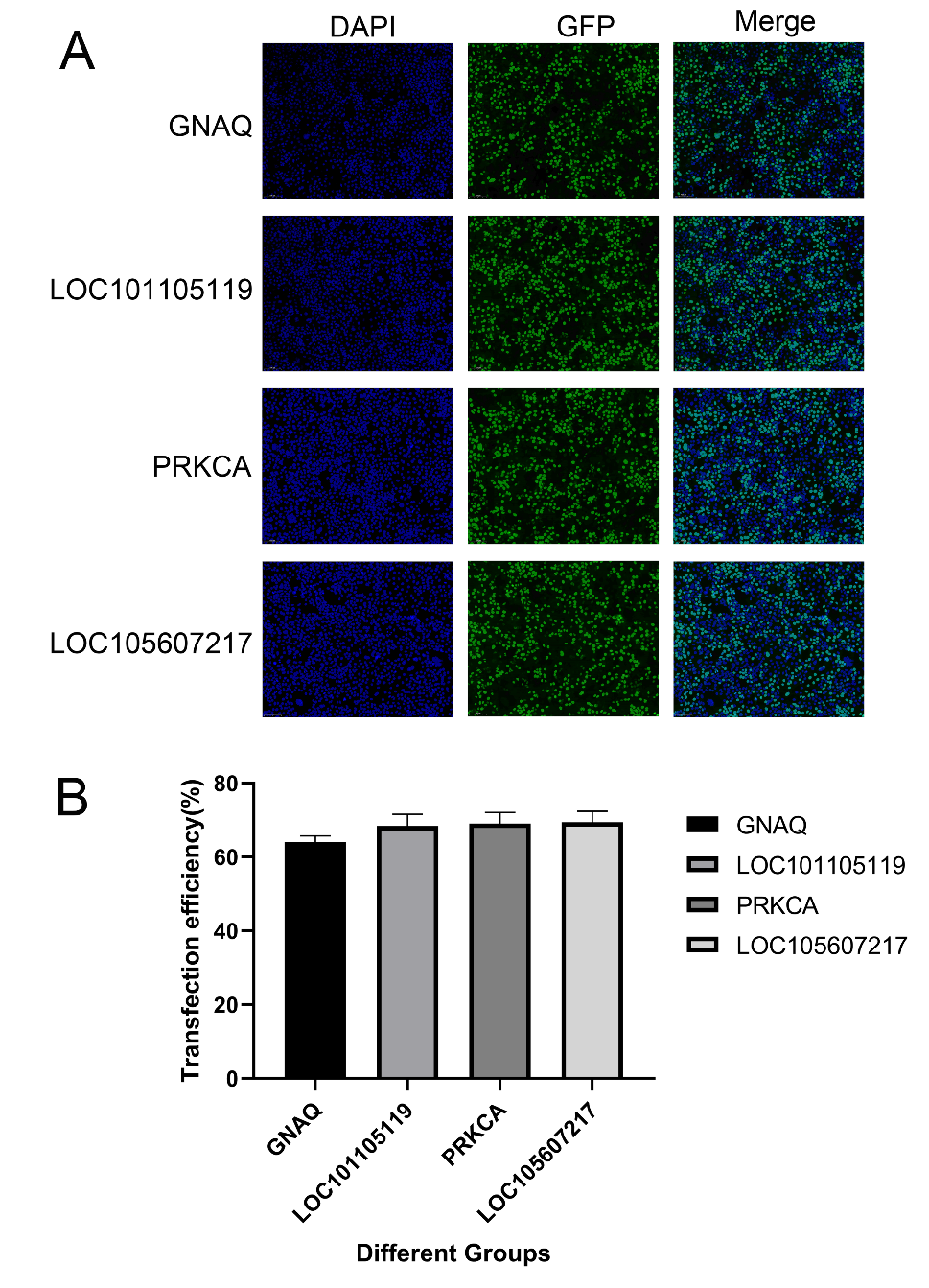


**Supplementary Figure 11.** Detection of cell transfection efficiency of overexpression vectors for *GNAQ*, LOC101105119, *PRKCA*, and LOC105607217. (**A**) HEK-293T cells were transfected with the pcDNA3.1 eukaryotic cell overexpression vectors for *GNAQ*, LOC101105119, *PRKCA*, and LOC105607217, which included a sequence encoding green fluorescent protein (GFP). Fluorescence microscopy images were captured after culturing the cells for 48 hours. (**B**) Bar graph of cell transfection efficiency in different groups.


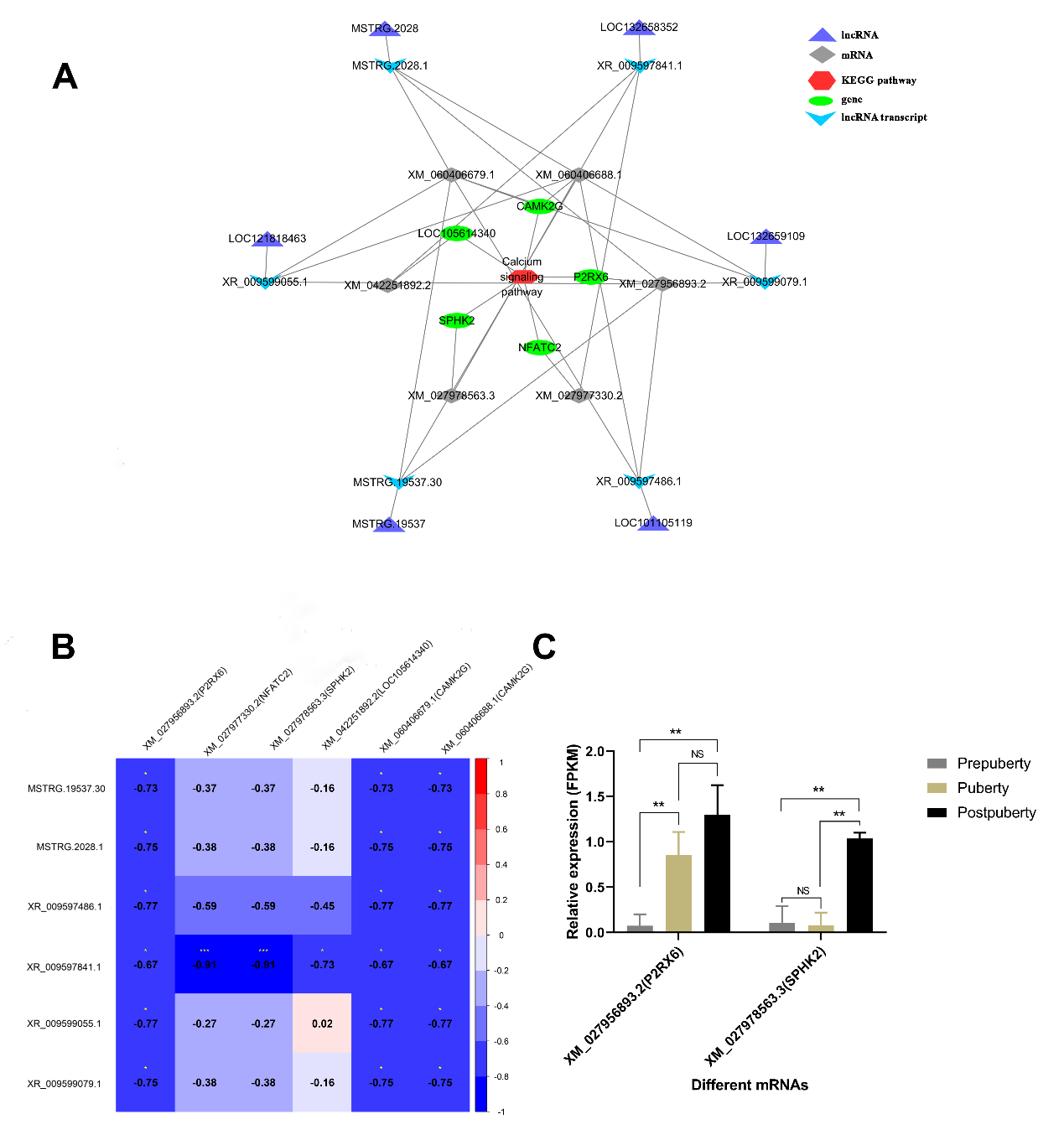


**Supplementary Figure 12.** Construction of a lncRNA-mRNA co-expression network associated with the calcium signaling pathway. (**A**) A lncRNA-mRNA co-expression network associated with the calcium signaling pathway. (**B**) Heat map illustrating the correlation between lncRNA and mRNA expression levels. (**C**) Bar graph depicting the expression levels of mRNAs in prepuberty, puberty, and postpuberty. * indicates *P* value < 0.05, ** indicate *P* value < 0.01, and NS indicate Not Significant.
